# Revisiting the Association of Pesticide Exposure and Parkinson's Disease: Systematic Review and Meta‐Analysis

**DOI:** 10.1002/tox.70041

**Published:** 2026-01-21

**Authors:** Pedro Henrique Passos da Silva, Jadson Silva Abreu, Rafael Sidônio Gibson Gomes, Hemengella Karyne Alves Oliveira, Artur F. S. Schuh, Ignacio F. Mata, Bruno Lopes Santos‐Lobato

**Affiliations:** ^1^ Laboratório de Neuropatologia Experimental Universidade Federal do Pará Belém Brazil; ^2^ Departamento de Farmacologia Universidade Federal do Rio Grande do Sul Porto Alegre Brazil; ^3^ Serviço de Neurologia, Hospital de Clínicas de Porto Alegre Porto Alegre Brazil; ^4^ Genomic Medicine, Lerner Research Institute, Cleveland Clinic Cleveland Ohio USA; ^5^ Serviço de Neurologia Hospital Ophir Loyola Belém Brazil

**Keywords:** herbicides, household, Parkinson's disease, pesticides, risk factors

## Abstract

The association between pesticide exposure and Parkinson's disease (PD) is substantial, but heterogeneity in methodology and lack of categorization according to the type of exposure and pesticide classes in previous meta‐analyses impair the interpretation of data. This study aims to update evidence of the association between pesticide exposure and PD. We conducted a systematic review and meta‐analysis of studies investigating associations between pesticide exposure and PD according to the type of pesticide exposure and pesticide class. We searched PubMed, EMBASE, and Web of Science until July 2024. Reviewers screened titles and abstracts. Afterward, reviewers reanalyzed the selection criteria and extracted the data based on the full paper. Meta‐analyses were conducted to assess the association between pesticide exposure and PD. A total of 124 studies were eligible. There is a lack of diversity in the populations represented and a high variability in methodology among the included studies. Considering only studies with any type of exposure, we found a positive association of PD with any pesticide class and herbicides. Occupational exposure was associated with PD for all pesticide classes except for fungicides. Exclusive household pesticide exposure was also associated with PD. Pesticide exposure remains a significant environmental risk factor for the development of PD, regardless of the type of exposure. Herbicides are the pesticide class with the most substantial evidence of association with the disease. Further studies with new methods of pesticide exposure measurement, innovative design studies, and the inclusion of underrepresented populations are still needed.

## 1 Introduction

The growing incidence of Parkinson's disease (PD) worldwide in the last decades [1] has led to a renewed effort to find an association between environmental risk factors associated with the condition, with a focus on pesticides. Since the first descriptions of early‐onset PD in two Italians with extensive pesticide use in 1986 [2] and two Brazilian rural workers caused by chronic exposure to the herbicide maneb in 1988 [3], many other epidemiological studies have explored the association, which has been confirmed by most of these works. Previous systematic reviews with meta‐analyses aggregated these data, showing that pesticide exposure increases the risk of developing PD [4, 5, 6, 7, 8, 9, 10, 11, 12, 13, 14].

However, the methodological heterogeneity of these studies has recently been highlighted [15], which may impair the generalizability of the results and weaken the level of evidence supporting the association between pesticide exposure and PD. In addition, most previous systematic reviews did not differentiate between studies based on the type of exposure (occupational or household) or pesticide classes (insecticides, herbicides, or fungicides).

While occupational exposure is more related to a rural setting, in which, farmworkers apply legalized pesticides on crops, usually with protective equipment, during specific periods of the year, the household exposure is urban and diffuse, without clear legislation about their commercialization and use, being a particular threat in developing regions [16]. Also, from a toxicological perspective, different pesticide classes may have distinct effects on neurodegenerative mechanisms associated with PD, which may vary depending on route of administration and dose. Thus, evaluating each pesticide type of exposure and class separately is needed for a more realistic perspective on their impact on human health.

Considering that the last systematic review including studies with evaluated occupational and household pesticide exposure was published in 2017 [11], and the growing number of publications on the topic in the last years, we conducted a systematic review and meta‐analysis of epidemiological studies on pesticide exposure and PD to update and improve the evidence of the association. More specifically, the present study aims to (1) estimate the association of pesticide exposure with PD through the odds ratio (OR), (2) compare the association with PD according to different types of exposure (occupational and household), and (3) compare the association with PD according to different pesticide classes (insecticides, herbicides, or fungicides).

## 2 Materials and Methods

### 2.1 Design

We conducted a systematic review with a meta‐analysis of original observational studies (cross‐sectional, case–control, and cohort) investigating associations between pesticide exposure and PD, published in English, Spanish, and Portuguese. We excluded studies that (1) assessed other parkinsonian conditions (such as atypical parkinsonism and dementia with Lewy bodies), (2) were conducted on animal models or in vitro, (3) were review studies, (4) were preprint articles, and (6) were duplicated articles. We followed the Preferred Reporting Items for Systematic Reviewers and Meta‐Analyses (PRISMA) 2020 guidelines to elaborate on this review. The PRISMA 2020 checklists are presented. The protocol was registered in the PROSPERO database (2024 CRD42024585463).

### 2.2 Search Strategy and Selection Process

Two independent investigators searched the literature in three databases (PubMed, EMBASE, and Web of Science) from inception to July 2024. The search strategy included terms for pesticide exposure and PD (see Data S1 for search term details).

All studies were imported into the Rayyan‐Intelligent Systematic Review software [17], which is used for recording decisions. In the first round, three investigators screened articles, blindly assessing titles and abstracts based on the inclusion and exclusion criteria. Disagreements were resolved by consensus with the assistance of a fourth reviewer.

For the second round, selected articles were randomly assigned to four investigators who evaluated the full texts for reanalyzing selection criteria and extracted the data using a structured online spreadsheet created specifically for this review in Google Sheets. Each study occupied a single row, and in columns the following items were reported: (1) first author's name, (2) year of publication, (3) geographical location of the studied population, (4) sample size in people with PD and controls, (5) sex and age at evaluation in people with PD and controls, (6) rural/urban origin of the studied population, (7) type of pesticide exposure (occupational/household/any exposure), (8) pesticide class evaluated (insecticide/herbicide/fungicide/any class), (9) number of people with PD exposed and nonexposed to pesticides, (10) number of controls exposed and nonexposed to pesticides. The items in the spreadsheet were derived from a priori research questions to support further meta‐analyses. For studies with issues deserving further discussion, the reports were reanalyzed by two reviewers to resolve disagreements.

We defined occupational pesticide exposure as any exposure to pesticides at work and household exposure as any exposure to pesticides in or around the home, according to the PD Risk Factor Questionnaire. However, when extracting data on the number of participants (with PD or controls) exposed or not exposed to pesticides in each study, we categorized the pesticide type of exposure according to the original definition from authors as “occupational” or “household”; if a categorization of exposure was not possible due to the lack of information in the study, it was labeled as “any exposure.”

For pesticide classes, we used the same categorization strategy based on the original definition from the authors into three classes, according to the PD Risk Factor Questionnaire: insecticides, herbicides, and fungicides. If a categorization of pesticide class into these three classes was not possible due to the lack of information, it was labeled as “any class.” Considering that most studies on the association between pesticides and PD do not describe exposure to other pesticide classes (e.g., rodenticides or acaricides), we restricted our categorization to three classes. To simplify further analyses, when the original study examined multiple pesticides within the same class (insecticides, herbicides, or fungicides), we extracted data from the product most commonly cited in other studies to represent exposure to that class.

The search strategy, selection process, and data extraction described above were conducted in accordance with PRISMA 2020 guidelines.

### 2.3 Quality Assessment

The quality of each selected study was evaluated using an instrument for exposure/outcome case–control studies in meta‐analyses, the Newcastle–Ottawa Quality Assessment Scale (NOS) [18]. This tool is based on eight items that assess the following themes: case definition, representativeness of cases, selection of controls, the definition of controls, comparability of cases and controls, ascertainment of exposure, a similar method of ascertainment for cases and controls, and nonresponse rate. Points were attributed to each item, and a score ranging from 0 to 9 was generated for each study. Scores 0–2 on the NOS indicate poor‐quality studies, 3–5 indicate fair quality, and 6–9 indicate high‐quality studies.

### 2.4 Data Analysis

To estimate the association between pesticide exposure and PD, meta‐analyses of original case–control studies were performed using R software version 4.0.4 (R Core Team 2018) with the package *metafor*. We used the random‐effects model with a continuity correction of 0.5 for studies with individuals without pesticide exposure in the PD or control group. The OR of developing PD among individuals with pesticide exposure was calculated using the restricted maximum likelihood (REML) estimator.

Egger's test was used to investigate publication bias. We also used Higgin's and Thompson's *I* ^2^ statistics to analyze heterogeneity between studies.

To identify potential sources of heterogeneity in the meta‐analysis, we conducted univariate meta‐regression analyses among studies included in the meta‐analysis that showed significant associations, using a mixed‐effects model. This analysis considered study quality and methodological factors, including rural/urban setting, number of participants, study origin (global subregion), method of exposure evaluation (questionnaire, geocoded, or pesticide measurement), and publication year.

Also, we performed sensitivity analyses to explore (I) the association of pesticide exposures with PD without studies from two leading research groups (Ritz group—USA—18 studies; Elbaz group—France—7 studies), aiming to reduce the overlap effect of including the same participant in different publications from the group, and (II) study quality through two subgroups (NOS < 6, poor and fair quality; NOS ≥ 6, high quality).

## 3 Results

### 3.1 Characteristics of Included Studies

**FIGURE 1:**
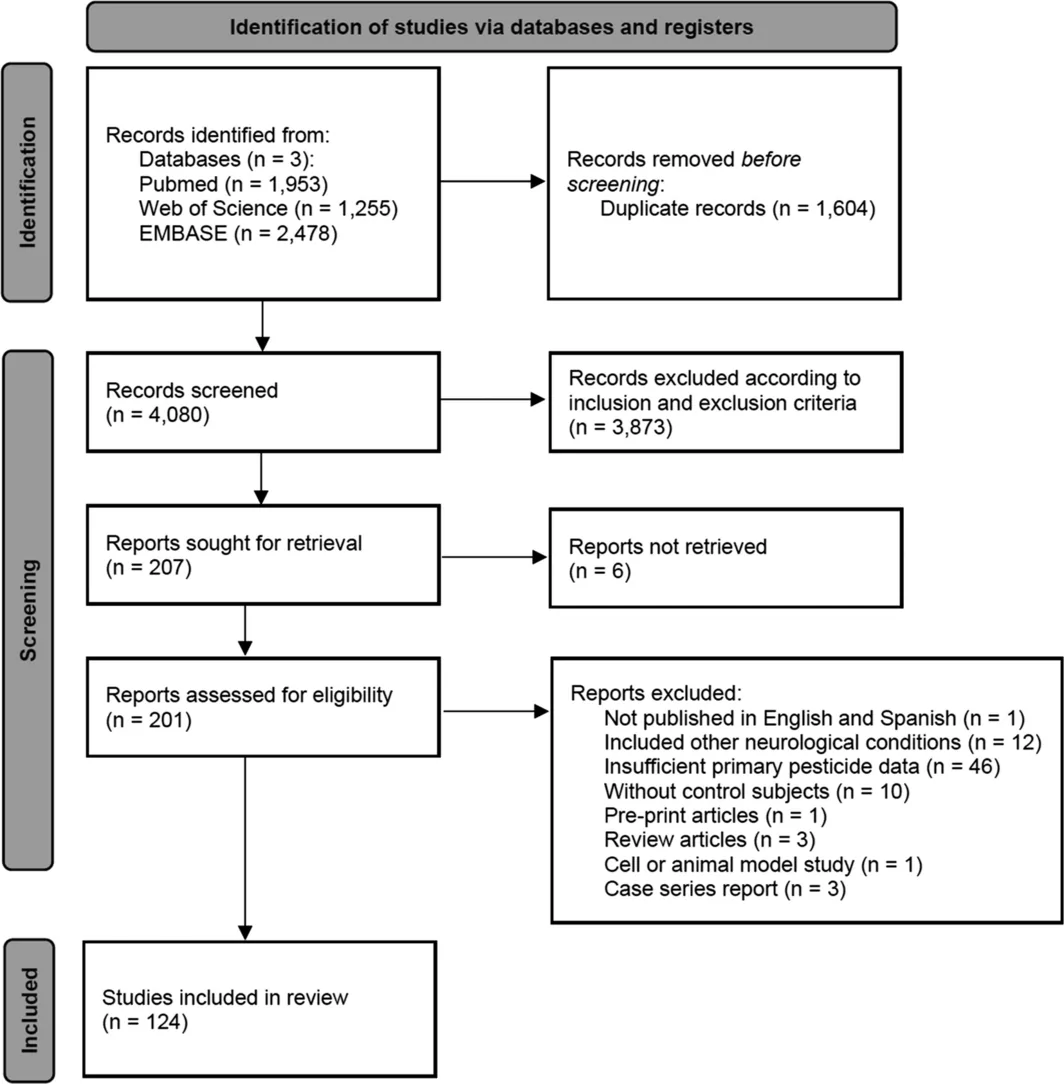
PRISMA 2020 flowchart.

We identified 5686 publications, of which 207 met the selection criteria for the first round. After the second round, 124 studies were eligible. The PRISMA 2020 flowchart (Figure 1) summarizes the step‐by‐step selection process. Table S1 shows the characteristics of the included studies and their respective references in Data S2.

Studies from 35 different countries were included. Most eligible studies were from North America (*n* = 55), with 48 studies from the United States. From Europe, 35 studies were selected, nine from France. Participation from other regions (Asia, *n* = 19; Central and South America, *n* = 6; Oceania, *n* = 6; Africa, *n* = 2) was more limited (Figure S1 and Table S2). According to the number of participants per region, the majority of recruited individuals were from North America, with Central and South America and Africa contributing the fewest. Regarding the sample size, only four studies enrolled more than 1000 people with PD. The quality assessment identified that most studies had fair quality (NOS 3–5; *n* = 85), with 21 studies classified as high quality (NOS ≥ 6).

Only 18 studies were population‐based (usually rural communities), and 38 studies recruited participants exclusively from rural regions. According to the study design, only 2 of the 124 studies were longitudinal cohorts, while the others were case–control or cross‐sectional studies. A total of 22 studies performed at least one sensitivity analysis. Movement disorder specialists confirmed the diagnosis of PD in 37 studies, general neurologists confirmed it in 67 studies, and the remaining 20 studies employed different strategies to confirm the PD diagnosis (non‐neurologist clinicians, self‐reported diagnosis, record linkage, and students). There was a slight predominance of males among the recruited participants, with males comprising 58.5% of individuals with PD and 53.4% of the control group. The mean age at evaluation of people with PD and controls was 67.0 and 66.3 years, respectively. The mean age at disease onset was 61.5 years.

Regarding pesticide data, exposure was quantified through environmental questionnaires in 102 studies, exposure estimates (geocoded data or job exposure matrix) in 14 studies, and exposure was based on measuring organochlorines in blood in seven studies. In one study, the data were based on a poisoning registry. Only 11 studies based on questionnaires tested the reliability of the instrument.

Exclusive occupational pesticide exposure was analyzed in 34 studies, exclusive household pesticide exposure in only five studies, and most evaluated both exposures (combined—any exposure, *n* = 58; segregated—occupational and household, *n* = 27). Pesticide classes were categorized in 61 studies, with the specific pesticides most frequently explored being paraquat (*n* = 16) and maneb (*n* = 8). Most studies (*n* = 82) defined pesticide exposure as any contact during participants' lifetimes, and 18 studies included definitions of high exposure for analyses, usually based on median values of duration and frequency of pesticide usage. We noted a marked variability in methodological procedures among studies on pesticide exposure measurement and analyses, as shown in Table 1.

**TABLE 1:** Variability in the categorization of methodological variables related to pesticide exposure among studies.

Pesticide exposure methodological variables	Possible categories
Measurement of pesticide exposure	Environmental questionnaire (usually homemade), geocoded exposure estimate, job exposure matrix estimate, registry of clinical poisoning, human biomonitoring in blood
Definition of pesticide exposure	Any exposure, exposure after early adolescence, minimum of months to years of exposure, at least years prior to PD onset
Definition of high pesticide exposure	Median of duration or frequency of usage, based on estimated cumulative exposure, usage higher than a specific frequency exposure, blood concentration higher than a specific percentile
Duration of pesticide exposure	Lifetime, per age range
Exposure categorization in age ranges	For each 10‐year age range, for each 20‐year age range (both after 16 years old)
Frequency of pesticide exposure	Number of uses per year, number of days per year, frequency categories (never, < 24 days per year, > 25 days per year; < 2 days per year, 2–11 days per year, > 1 use per month; never, 1–5 days per year, 6–10 days per year, 11–30 days per year, > 30 days per year)
Statistical analysis	Logistic regression, Cox regression, Poisson regression, simple odds ratio calculation
Covariables in regression analysis models	Sex, smoking, education, family history, genetic data, head trauma, country, rural origin, urbanization level, socioeconomic status, ethnicity, caffeine consumption, alcohol consumption, well water consumption, marital status, use of illicit drugs, Mini‐Mental State Examination, body mass index, stroke, and hypertension

### 3.2 Association Between Any Type of Pesticide Exposure and PD

**FIGURE 2:**
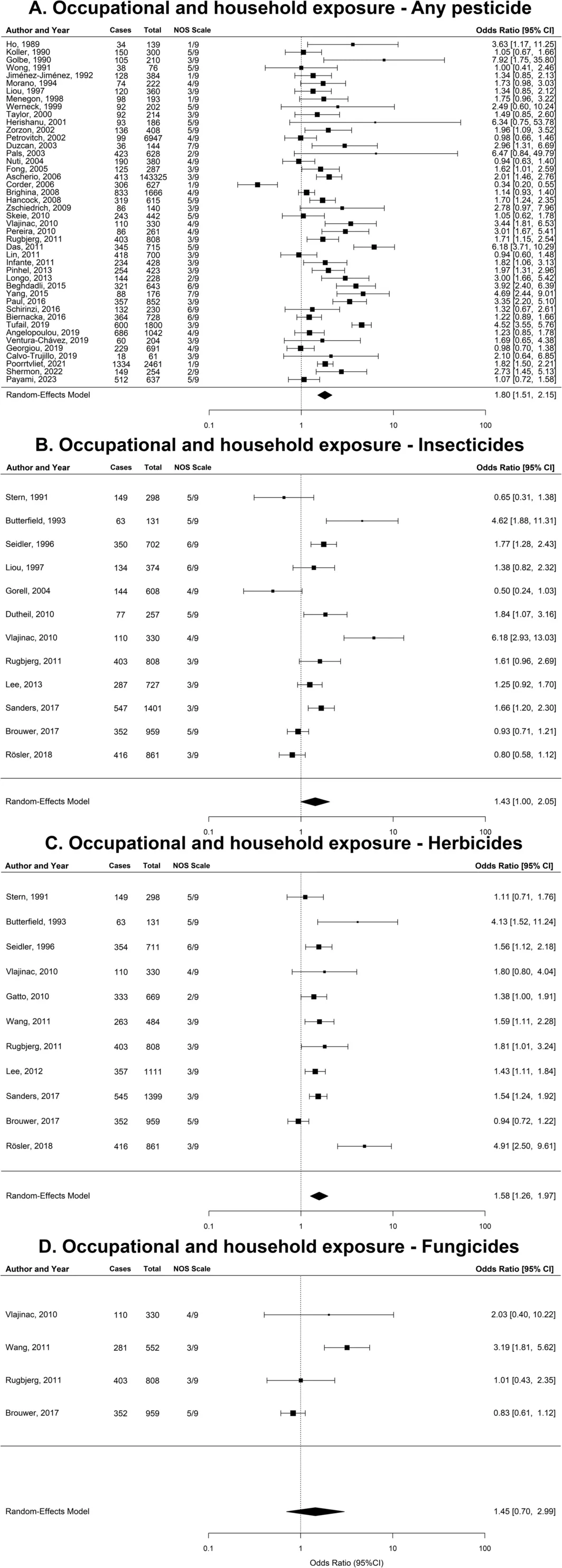
Meta‐analyses of the association of occupational and household pesticide exposure and Parkinson's disease. (A) Meta‐analysis for any pesticide class; (B) meta‐analysis for insecticides; (C) meta‐analysis for herbicides; (D) meta‐analysis for fungicides. Boxes are proportional to study sizes. A diamond symbol represents the pooled result. CI, confidence interval; NOS, Newcastle–Ottawa Scale score.

Considering any type of pesticide exposure (occupational or household), the meta‐analysis showed that the use of any pesticide class (*n* = 44 studies) during lifetime increased the association with PD (OR = 1.80; 95% CI = 1.51–2.14) (Table 2 and Figure 2A), with high heterogeneity among studies (*I* ^2^ statistics = 83.4%). For herbicides (*n* = 11), the meta‐analyses showed that the use of this pesticide class during a lifetime increased the association with PD (OR = 1.58; 95% CI = 1.26–1.97) (Table 2 and Figure 2B), with high heterogeneity (*I* ^2^ statistics = 72.4%). For fungicides (*n* = 4; number of participants = 2649), the meta‐analysis showed no association with PD (OR = 1.45; 95% CI = 0.70–2.99) (Table 2 and Figure 2D), but for insecticides (*n* = 12), the meta‐analyses showed a trend of increasing the association with PD (OR = 1.43; 95% CI = 1.00–2.05, *p* = 0.0524) (Table 2 and Figure 2C), both with high heterogeneity (fungicides: *I* ^2^ statistics = 78.5%; insecticides: *I* ^2^ statistics = 87.7%). According to Egger's test, no publication bias was detected among the studies.

**TABLE 2:** Meta‐analysis of the association between pesticide exposure and Parkinson's disease.

Exposure	Pesticide class	*K*	*n*	Random‐effects model			Heterogeneity	Egger's test
				OR	95% CI	*p*	*I* ^2^	
Any type	Any pesticides	44	171 767	**1.80**	**1.51–2.14**	**< 0.0001**	83.45	*p* = 0.30 (*b* = 0.35)
	Insecticides	12	7456	1.43	1.00–2.05	0.0524	87.75	*p* = 0.30 (*b* = −0.12)
	Herbicides	11	7761	**1.58**	**1.26–1.97**	**< 0.0001**	72.49	*p* = 0.06 (*b* = −0.03)
	Fungicides	4	2649	1.45	0.70–2.99	0.3166	78.56	*p* = 0.50 (*b* = −0.33)
Occupational	Any pesticides	40	104 532	**1.46**	**1.27–1.68**	**< 0.0001**	71.37	*p* = 0.15 (*b* = 0.11)
	Insecticides	20	237 515	**1.46**	**1.13–1.88**	**0.0033**	75.36	*p* = 0.13 (*b* = −0.10)
	Herbicides	21	239 157	**1.27**	**1.08–1.50**	**0.0045**	59.37	*p* = 0.005 (*b* = −0.19)
	Fungicides	15	236 158	1.25	0.99–1.60	0.0656	64.71	*p* = 0.19 (*b* = −0.06)
Household	Any pesticides	23	255 056	**1.21**	**1.06–1.39**	**0.0058**	75.15	*p* = 0.87 (*b* = 0.16)
	Insecticides	8	4209	1.29	0.98–1.70	0.0699	72.29	*p* = 0.73 (*b* = 0.42)
Any type	Paraquat (herbicide)	6	5050	**1.34**	**1.13–1.60**	**0.0007**	49.92	*p* = 0.98 (*b* = 0.28)
Occupational	Paraquat (herbicide)	8	233 208	1.21	0.96–1.53	0.1061	72.31	*p* = 0.053 (*b* = −0.27)

*Note:* Bold text indicates statistically significant associations.

Abbreviations: *b*, Egger's test intercept; CI, confidence interval; *I* ^2^, Higgin's and Thompson's *I* ^2^ statistic; *K*, total number of studies on a pesticide exposure; *n*, total number of participants (counts) included in studies on a pesticide exposure; OR, odds ratio.

According to the meta‐regression analyses for any pesticide class, insecticides, and herbicides, study quality, rural/urban setting, number of participants, study global subregion, method of exposure estimate, and the publication year did not influence heterogeneity, except for the rural/urban setting in any pesticide class and global subregion in herbicides. However, lower study quality, older published studies, small sample sizes, and global subregions had higher effect sizes in heterogeneity for fungicides.

### 3.3 Association Between Occupational Pesticide Exposure and PD

**FIGURE 3:**
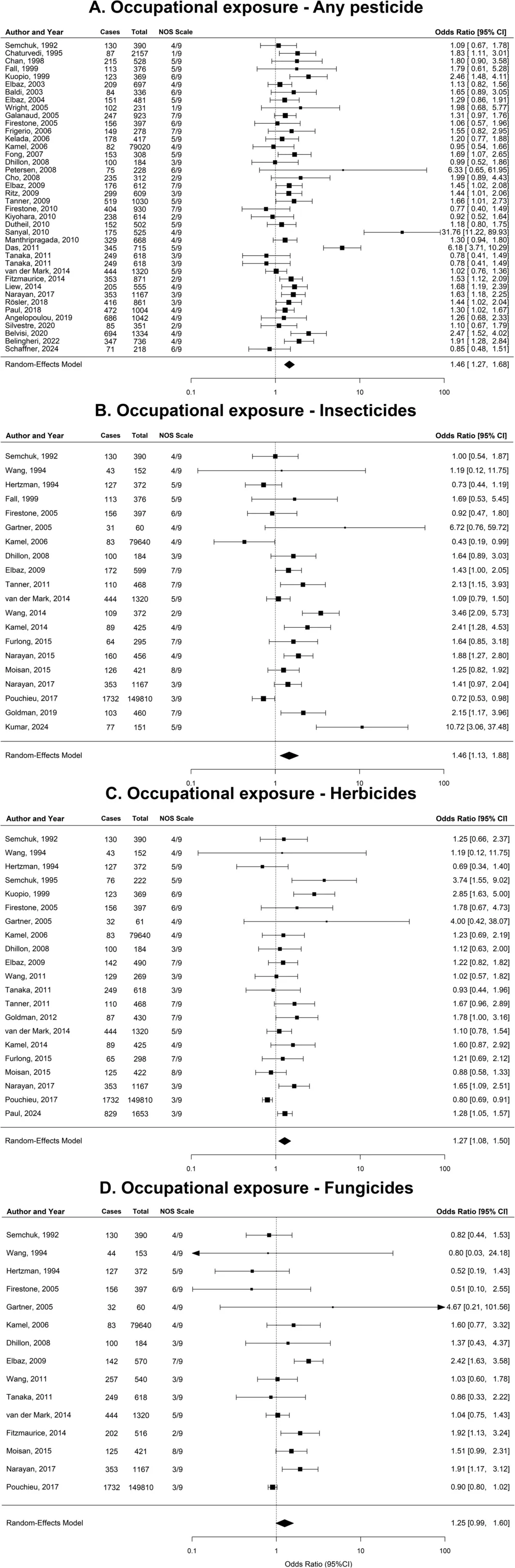
Meta‐analyses of the association of only occupational pesticide exposure and Parkinson's disease. (A) Meta‐analysis for any pesticide class, (B) meta‐analysis for insecticides, (C) meta‐analysis for herbicides, (D) meta‐analysis for fungicides. Boxes are proportional to study sizes. A diamond symbol represents the pooled result. CI, confidence interval; NOS, Newcastle–Ottawa Scale score.

Considering only occupational pesticide exposure, the meta‐analysis showed that the use of any pesticide class (*n* = 40 studies) during lifetime increased the association with PD (OR = 1.46; 95% CI = 1.27–1.68) (Table 2 and Figure 3A), with high heterogeneity among studies (*I* ^2^ statistics = 71.3%). For insecticides (*n* = 20) and herbicides (*n* = 21), the meta‐analysis showed that exposure to these pesticide classes during the lifetime increased the association with PD (insecticides: OR = 1.46; 95% CI = 1.13–1.88; herbicides: OR = 1.27; 95% CI = 1.08–1.50) (Table 2 and Figure 3B,C), with high and moderate heterogeneity, respectively (insecticides: *I* ^2^ statistics = 75.3%; herbicides: *I* ^2^ statistics = 59.3%). For fungicides (*n* = 15), the meta‐analyses showed a trend toward statistical significance for the association with PD (OR = 1.25; 95% CI = 0.99–1.60, *p* = 0.0656) (Table 2 and Figure 3D) with high heterogeneity (*I* ^2^ statistics = 64.7%). According to Egger's test, a publication bias was found among studies on occupational herbicide exposure.

According to the meta‐regression analyses for any pesticide class, study quality, rural/urban setting, number of participants, study global subregion, method of exposure estimate, and publication year did not influence heterogeneity. However, the number of participants and publication year had some effect on heterogeneity for studies on insecticides and herbicides.

### 3.4 Association Between Household Pesticide Exposure and PD

**FIGURE 4:**
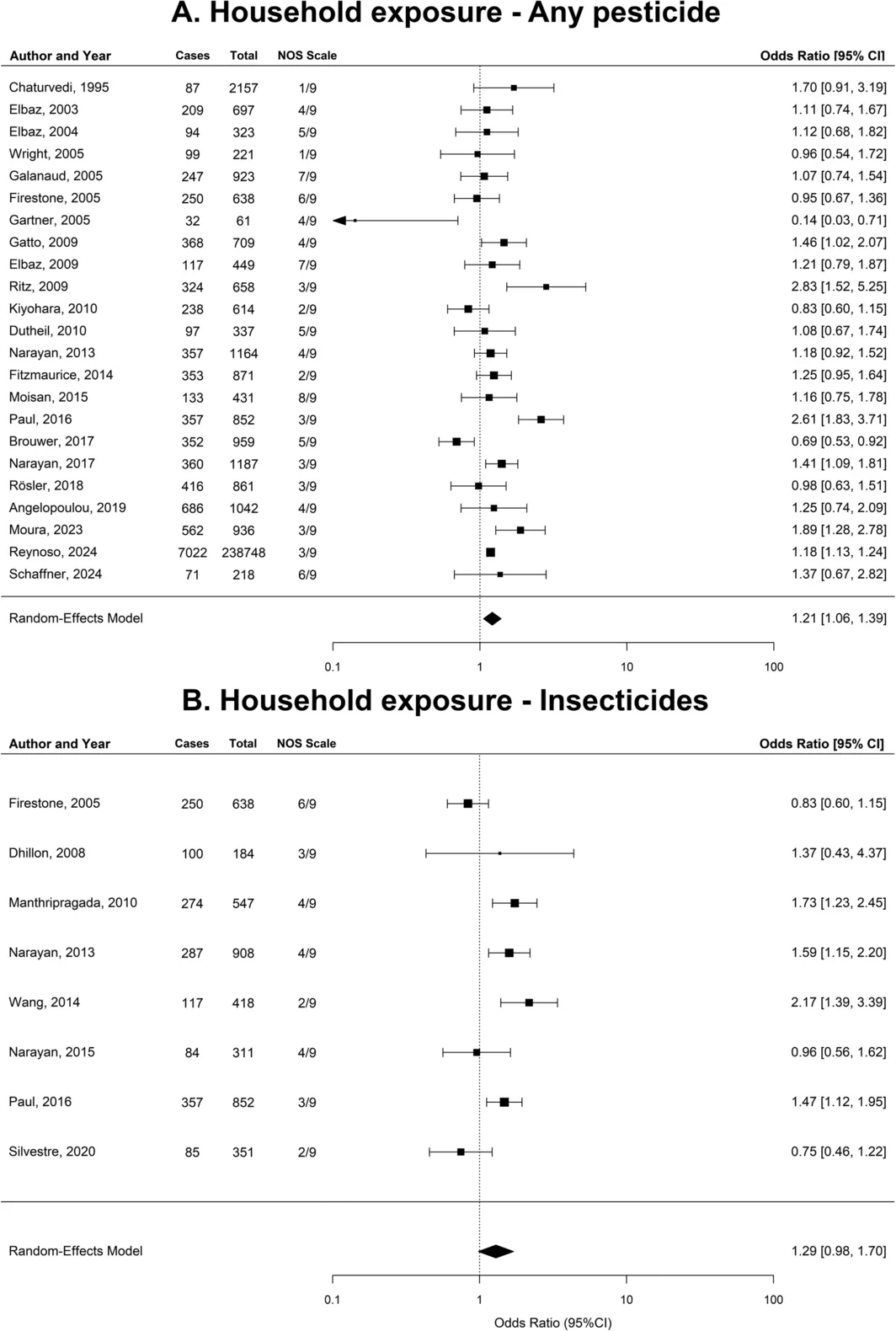
Meta‐analyses of the association of only household pesticide exposure and Parkinson's disease. (A) Meta‐analysis for any pesticide class, (B) meta‐analysis for insecticides. Boxes are proportional to study sizes. A diamond symbol represents the pooled result. CI, confidence interval; NOS, Newcastle–Ottawa Scale score.

Considering only household pesticide exposure, the meta‐analysis showed that the use of any pesticide class (*n* = 23 studies) during lifetime increased the association with PD (OR = 1.21; 95% CI = 1.06–1.39) (Table 2 and Figure 4A), with high heterogeneity among studies (*I* ^2^ statistics = 75.1%). For insecticides (*n* = 8; number of participants = 4209), the meta‐analysis showed a trend toward statistical significance for the association with PD (OR = 1.29; 95% CI = 0.98–1.70, *p* = 0.0699) (Table 2 and Figure 4B), with high heterogeneity (*I* ^2^ statistics = 72.2%). The limited number of studies on household pesticide exposure involving herbicides and fungicides did not permit meta‐analyses. According to Egger's test, no publication bias was detected among the studies.

According to the meta‐regression analyses for any pesticide class or insecticide, study quality, rural/urban setting, number of participants, study global subregion, method of exposure estimate, and publication year did not influence heterogeneity (Table S3).

### 3.5 Association Between Paraquat Exposure and PD

Considering only the use of paraquat, the meta‐analysis showed that, when occupational and household use were not differentiated (*n* = 6 studies; number of participants = 5050), the exposure to the product during lifetime increased the association with PD (OR = 1.34; 95% CI = 1.13–1.60) (Table 2 and Figure S2A), with moderate heterogeneity among studies (*I* ^2^ statistics = 49.9%). For a strict occupational paraquat exposure (*n* = 8; number of participants = 233 208), the meta‐analysis showed no association with PD (OR = 1.21; 95% CI = 0.96–1.53) (Table 2 and Figure S2B) with high heterogeneity (*I* ^2^ statistics = 72.3%). The small number of studies on household paraquat exposure did not allow meta‐analyses. According to Egger's test, no publication bias was detected among the studies.

According to the meta‐regression analyses for any type of paraquat exposure, study quality and global subregions were found to affect heterogeneity. For occupational paraquat exposure, the number of participants and the global subregion influenced the heterogeneity.

### 3.6 Sensitivity Analyses

According to leading research groups, after excluding studies from the Ritz group (*n* = 18), the associations between any household exposure and any paraquat exposure with PD were not significant (any household exposure: *p* = 0.2542; any paraquat exposure: *p* = 0.7127). Excluding studies from the Elbaz group (France, *n* = 7), no relevant association modifications were observed (Table S4). According to study quality, analyses were impaired due to a low number of high‐quality studies (NOS ≥ 6). Most associations remained unchanged for studies with poor or fair quality, as well as those with high quality (Table S5).

## 4 Discussion

The present systematic review provides additional evidence that any pesticide exposure during the lifetime, regardless of the type of exposure, may be associated with an increased risk of PD by approximately 80%. A total of 23 studies were published after the last most thorough systematic review on the topic [11], most of which have not yet been included in a systematic review. This effect is particularly relevant for herbicides. Both specific occupational and household exposures are related to this effect. The herbicide paraquat is the product with the most substantial evidence of association with PD. Studies exhibit high methodological heterogeneity, primarily due to variations in pesticide measurement and analysis. These results align with previous systematic reviews and meta‐analyses on the impact of pesticide exposure and the risk of developing PD [4, 5, 6, 7, 8, 9, 10, 11, 12, 13, 14].

Occupational exposure was the most extensively explored aspect of pesticide use in our systematic review, likely due to its agricultural applications. These products may affect nearly 1 billion rural workers worldwide [19], and the frequent health monitoring of these exposed populations living in rural regions with intense agricultural activity is justified. It may explain the high number of studies from two research groups in the United States and France, which analyzed rural counties in the Central Valley of California and individuals affiliated with the French *Mutualité Sociale Agricole*, respectively. According to our sensitivity analyses, the potential overrepresentation of studies from these rural settings in our meta‐analyses affected the association with PD.

However, no previous systematic review analyzed the overall association between household pesticide exposure and PD. It is worth noting that nearly 4.4 billion people live in cities globally, and around 50%–90% of urban individuals have reported using pesticides during their lifetime [20]. Household pesticide use may pose a risk to human health, particularly in low‐ and middle‐income countries, due to improper handling without the use of personal protective equipment and an informal market [21]. According to our results, household pesticide exposure has a similar magnitude of association with PD to occupational exposure, suggesting that further studies on this type of exposure are needed [22].

Herbicides were the pesticide class most associated with PD in studies involving any type of exposure, particularly occupational exposure, with a high number of participants (*n* > 200 000). Household herbicide exposure description was rare. Previous meta‐analyses have shown divergent results for herbicides [6, 9]. For occupational exposure, herbicides are the world's most widely used pesticide class in agriculture [23]. An earlier study performed a toxicogenomic analysis and found an association between PD and a list of 70 chemical products, including 14 herbicides [24]. One of these herbicides is glyphosate, the most widely used herbicide worldwide [25].

Only occupational exposure to insecticides had a significant association with PD. Regarding household insecticide exposure, only a trend of association with PD was seen, possibly due to the low total number of participants. Previous meta‐analyses have reported a positive association between any type of insecticide exposure [6], but not with occupational use [6, 9]. Also, there was no association between fungicide exposure and PD in our study and previous meta‐analyses [6, 9].

The low number of studies with household and specific pesticide class exposure may be due to data being mainly based on environmental questionnaires with high heterogeneity. Analyses that do not discriminate between the types of pesticide use or distinct exposure to herbicides, insecticides, and fungicides may impair the interpretation of results [15]. Considering this, area‐based geocoding techniques for estimating pesticide exposure may be viable alternatives to questionnaire‐based studies, especially in rural areas with well‐characterized land‐use data and pesticide use reports [26].

### 4.1 Geographic Representation

Despite the high number of studies, there is a notable lack of diversity in the countries represented in these analyses. There is an overrepresentation of people from the United States, as approximately 40% of all included studies were from this country (with a total of 328 408 participants from the United States). In our systematic review, the two most populous countries, India and China, were represented by only four and two studies, respectively. These six studies recruited a total of fewer than 2000 participants. Furthermore, the populations of the other top 10 most populous countries were not represented in the present study, including Indonesia, Pakistan, Nigeria, Bangladesh, and the Russian Federation.

The participation of some global regions, such as Latin America and the Caribbean, sub‐Saharan Africa, the Middle East and North Africa, Eastern Europe, Central Asia, and East and Southeast Asia, is markedly underrepresented. A similar pattern of underrepresentation of people from the Global South in PD research has been previously described in genetic studies [27]. Considering occupational pesticide exposure, agricultural production is the main economic activity of many of these underrepresented countries. In addition, the legal regulation of pesticide use in agriculture in low‐ and middle‐income countries may be more permissive, allowing products with health issues [28].

Finally, a recent report from the Food and Agriculture Organization identified the top 10 countries with the highest pesticide use in 2023 (in descending order): Brazil, the United States, Indonesia, Argentina, China, Australia, Viet Nam, the Russian Federation, Canada, and France [23]. According to our systematic review, no studies from four of these countries (Indonesia, Argentina, Viet Nam, and the Russian Federation) were included, showing an underrepresentation of countries with high agricultural pesticide use. Brazil, the country with the highest pesticide use in agriculture, contributed only four studies on occupational pesticide exposure. Overall, these issues in the geographic representation of countries may limit the generalizability of our results. Furthermore, the lack of studies from already cited underrepresented regions and the low number of studies that categorized the type of exposure and specific pesticide class may also impair the generalizability of findings.

### 4.2 Methodological Limitations

The present systematic review had limitations. One of them was that the definitions of pesticide exposure types and pesticide classes, based on the original study authors' descriptions, may not be precise and were not evident in the text of some studies. However, for studies without a clear definition of the type of pesticide exposure and pesticide classes, we used broad categorizations (“any exposure” and “any class”).

Thus, previous meta‐analyses and the present study indicate the same level of association between pesticide exposure and PD. However, we have to question the significance and relevance of these results in light of the methodological flaws in the original studies.

First, these studies were strongly based on data from environmental questionnaires for lifetime pesticide exposure, which are very heterogeneous among studies and subject to inaccuracy and recall bias, a form of differential misclassification bias influenced by time interval since exposure, personal characteristics of participants (age, education, socioeconomic status), the significance of the past event for individuals, social desirability of exposure, interviewing technique, and differential recall between cases and controls [29].

Second, excluding two cohort studies and seven cross‐sectional studies (measurement of organochlorines in the blood at evaluation), all other 115 studies had a case–control design (where PD and control status served as the study inclusion criteria, and a retrospective analysis was conducted to determine which individuals were exposed to pesticides in the past) [30]. As is known, observational retrospective studies cannot be used to establish a causal link between exposure and outcome but may infer an association or correlation between them [31, 32].

### 4.3 Future Directions

After dozens of published works on the theme since 1989, we can now argue that all evidence of an association between pesticide exposure and PD that could be inferred from this classic study design (case–control studies with environmental questionnaires) has already been extracted. But how do we increase the evidence of causality?

Recently, a review examined the causal link between various environmental risk factors and PD, including exposure to pesticides. According to the Bradford Hill criteria, the authors proposed that there is good evidence for an association between general pesticide exposure and PD in seven of the nine criteria. The authors proposed actions for three strategies (epidemiological, pathophysiological studies, and policy changes) to fill evidence gaps, including new studies with biomarkers for pesticide exposure [28]. However, the development of new biomarkers—such as cumulative pesticide residue detection, nuclear, mitochondrial, and epigenetic markers, as well as metabolomic and neuroinflammatory profiles—is crucial, given the current lack of reliable methods to quantify long‐term exposure to most pesticides [22].

In conclusion, this systematic review updated the evidence that pesticide exposure is associated with an increased risk of PD across different types of exposure. The strength of this association may depend on the pesticide class, with herbicides showing stronger associations. However, evidence derived from classic case–control studies that rely on environmental questionnaires remains limited in its ability to support causal inference. Future research should prioritize improved methods for pesticide exposure assessment and more innovative study designs, including longitudinal exposure records, biomarker‐based analyses, and area‐based geocoding approaches in underrepresented populations.

## Funding

The authors have nothing to report.

## Conflicts of Interest

Artur F.S. Schuh has received grants from the Michael J. Fox Foundation (MJFF), the Aligning Science Across Parkinson's Global Parkinson Genetic Program (ASAP‐GP2), Brazilian National Council for Scientific and Technological Development (CNPq—Brazil), Fapergs (Brazil), and honoraria from MDS. Ignacio F. Mata has received grants from the Stanley Fahn Junior Faculty Award and an International Research Grants Program award, both from the Parkinson's Foundation, and a research grant from the American Parkinson's Disease Association. He is currently funded by the National Institutes of Health, MJFF, and the ASAP‐GP2. Bruno Lopes Santos‐Lobato has received a grant from the CNPq—Brazil. The other authors declare no conflicts of interest.

## Supporting information


**Data S1:** Search strategy term detail.
**Data S2:** References from selected studies for the present study.

**Figure S1:** Selected studies count by countries/regions. The number of included studies per country was indicated on the map for countries with more than two studies.

**Figure S2:** Meta‐analyses of the association between paraquat exposure and Parkinson's disease. (A) Meta‐analysis for occupational and household paraquat exposure. (B) Meta‐analysis for occupational paraquat exposure. Boxes are proportional to study sizes. A diamond symbol represents the pooled result. CI, confidence interval; NOS, Newcastle–Ottawa Scale score.

**Table S1:** Characteristics of studies selected in a meta‐analysis for the association of pesticide exposure and Parkinson's disease.

**Table S2:** Number of participants from selected studies per global subregion.

**Table S3:** Influence of methodological variables as sources of heterogeneity among studies included in meta‐analyses showing the association between pesticide exposure and risk of developing Parkinson's disease.

**Table S4:** Meta‐analysis of the association among pesticide exposure and Parkinson's disease after excluding studies from leading research groups.

**Table S5:** Meta‐analysis of the association among pesticide exposure and Parkinson's disease according to study quality by the Newcastle–Ottawa Scale.

## Data Availability

The data that support the findings of this study are openly available in Zenodo at https://zenodo.org/, reference number 10.5281/zenodo.15033944.
