# Supplementary material for: Revisiting the Association of Pesticide Exposure and Parkinson's Disease: Systematic Review and Meta‐Analysis
Source: Environ Toxicol. 2026 Jan 21;41(10):875–88. doi: 10.1002/tox.70041 (PMC13537366; doi:10.1002/tox.70041)
Supplement: Supplementary file 1 [file TOX-41-875-s002.docx]

**Supplementary Materials**

**Data S1.** Search strategy term detail

PubMed:

"Pesticides"[All Fields] AND ("Parkinson disease"[MeSH Terms] OR ("Parkinson"[All Fields] AND "Disease"[All Fields]) OR "Parkinson disease"[All Fields] OR ("Parkinson's"[All Fields] AND "Disease"[All Fields]) OR "PD"[All Fields])) NOT ("Review"[Publication Type] OR "Editorial"[Publication Type]);

EMBASE:

('parkinson disease'/exp OR 'parkinson disease') AND ('pesticide'/exp OR 'pesticide')) AND ('article'/it OR 'article in press'/it OR 'conference paper'/it OR 'short survey'/it);

Web of Science:

parkinson** disease (Topic) AND pesticide* (Topic) NOT Review OR Editorial Material OR Meeting Abstract OR Book Chapter OR Note OR Proceedings Paper (Document Type).

**Data S2.** References from selected studies for the present study

e1.	[Ho SC, Woo J, Lee CM. Epidemiologic study of Parkinson’s disease in Hong Kong. Neurology 1989;39(10):1314–1318.](http://paperpile.com/b/9ckeIm/hI15)

e2.	[Koller W, Vetere-Overfield B, Gray C, et al. Environmental risk factors in Parkinson’s disease. Neurology 1990;40(8):1218–1221.](http://paperpile.com/b/9ckeIm/iaTS)

e3.	[Golbe LI, Farrell TM, Davis PH. Follow-up study of early-life protective and risk factors in Parkinson’s disease. Mov Disord 1990;5(1):66–70.](http://paperpile.com/b/9ckeIm/hAgD)

e4.	[Wong GF, Gray CS, Hassanein RS, Koller WC. Environmental risk factors in siblings with Parkinson’s disease. Arch Neurol 1991;48(3):287–289.](http://paperpile.com/b/9ckeIm/YEEf)

e5.	[Stern M, Dulaney E, Gruber SB, et al. The epidemiology of Parkinson’s disease. A case-control study of young-onset and old-onset patients. Arch Neurol 1991;48(9):903–907.](http://paperpile.com/b/9ckeIm/z8qm)

e6.	[Semchuk KM, Love EJ, Lee RG. Parkinson’s disease and exposure to agricultural work and pesticide chemicals. Neurology 1992;42(7):1328–1335.](http://paperpile.com/b/9ckeIm/5FPS)

e7.	[Jiménez-Jiménez FJ, Mateo D, Giménez-Roldán S. Exposure to well water and pesticides in Parkinson’s disease: a case-control study in the Madrid area. Mov Disord 1992;7(2):149–152.](http://paperpile.com/b/9ckeIm/VGSh)

e8.	[Butterfield PG, Valanis BG, Spencer PS, et al. Environmental antecedents of young-onset Parkinson’s disease. Neurology 1993;43(6):1150–1158.](http://paperpile.com/b/9ckeIm/G7m7)

e9.	[Wang FL, Semchuk KM, Love EJ. Reliability of environmental and occupational exposure data provided by surrogate respondents in a case-control study of Parkinson’s disease. J Clin Epidemiol 1994;47(7):797–807.](http://paperpile.com/b/9ckeIm/M6ZH)

e10.	[Hertzman C, Wiens M, Snow B, et al. A case-control study of Parkinson’s disease in a horticultural region of British Columbia. Mov Disord 1994;9(1):69–75.](http://paperpile.com/b/9ckeIm/CiQC)

e11.	[Morano A, Jiménez-Jiménez FJ, Molina JA, Antolín MA. Risk-factors for Parkinson’s disease: case-control study in the province of Cáceres, Spain. Acta Neurol Scand 1994;89(3):164–170.](http://paperpile.com/b/9ckeIm/tblH)

e12.	[Chaturvedi S, Ostbye T, Stoessl AJ, et al. Environmental exposures in elderly Canadians with Parkinson’s disease. Can J Neurol Sci 1995;22(3):232–234.](http://paperpile.com/b/9ckeIm/OHud)

e13.	[Semchuk KM, Love EJ. Effects of agricultural work and other proxy-derived case-control data on Parkinson’s disease risk estimates. Am J Epidemiol 1995;141(8):747–754.](http://paperpile.com/b/9ckeIm/PmPX)

e14.	[Seidler A, Hellenbrand W, Robra BP, et al. Possible environmental, occupational, and other etiologic factors for Parkinson’s disease: a case-control study in Germany. Neurology 1996;46(5):1275–1284.](http://paperpile.com/b/9ckeIm/xjZt)

e15.	[Liou HH, Tsai MC, Chen CJ, et al. Environmental risk factors and Parkinson’s disease: a case-control study in Taiwan. Neurology 1997;48(6):1583–1588.](http://paperpile.com/b/9ckeIm/66AW)

e16.	[McCann SJ, LeCouteur DG, Green AC, et al. The epidemiology of Parkinson’s disease in an Australian population. Neuroepidemiology 1998;17(6):310–317.](http://paperpile.com/b/9ckeIm/FBO9)

e17.	[Gorell JM, Johnson CC, Rybicki BA, et al. The risk of Parkinson’s disease with exposure to pesticides, farming, well water, and rural living. Neurology 1998;50(5):1346–1350.](http://paperpile.com/b/9ckeIm/hRSB)

e18.	[Chan DK, Woo J, Ho SC, et al. Genetic and environmental risk factors for Parkinson’s disease in a Chinese population. J Neurol Neurosurg Psychiatry 1998;65(5):781–784.](http://paperpile.com/b/9ckeIm/ywFD)

e19.	[Menegon A, Board PG, Blackburn AC, et al. Parkinson’s disease, pesticides, and glutathione transferase polymorphisms. Lancet 1998;352(9137):1344–1346.](http://paperpile.com/b/9ckeIm/bAMt)

e20.	[Werneck AL, Alvarenga H. Genetics, drugs and environmental factors in Parkinson’s disease. A case-control study. Arq Neuropsiquiatr 1999;57(2B):347–355.](http://paperpile.com/b/9ckeIm/WDvw)

e21.	[Fall PA, Fredrikson M, Axelson O, Granérus AK. Nutritional and occupational factors influencing the risk of Parkinson’s disease: a case-control study in southeastern Sweden. Mov Disord 1999;14(1):28–37.](http://paperpile.com/b/9ckeIm/xxpG)

e22.	[Kuopio AM, Marttila RJ, Helenius H, Rinne UK. Environmental risk factors in Parkinson’s disease. Mov Disord 1999;14(6):928–939.](http://paperpile.com/b/9ckeIm/uXmS)

e23.	[Taylor MC, Le Couteur DG, Mellick GD, Board PG. Paraoxonase polymorphisms, pesticide exposure and Parkinson’s disease in a Caucasian population. J Neural Transm (Vienna) 2000;107(8-9):979–983.](http://paperpile.com/b/9ckeIm/nHVe)

e24.	[Herishanu YO, Medvedovski M, Goldsmith JR, Kordysh E. A case-control study of Parkinson’s disease in urban population of southern Israel. Can J Neurol Sci 2001;28(2):144–147.](http://paperpile.com/b/9ckeIm/UWVN)

e25.	[Zorzon M, Capus L, Pellegrino A, et al. Familial and environmental risk factors in Parkinson’s disease: a case-control study in north-east Italy. Acta Neurol Scand 2002;105(2):77–82.](http://paperpile.com/b/9ckeIm/7qMK)

e26.	[Petrovitch H, Ross GW, Abbott RD, et al. Plantation work and risk of Parkinson disease in a population-based longitudinal study. Arch Neurol 2002;59(11):1787–1792.](http://paperpile.com/b/9ckeIm/FWvk)

e27.	[Baldi I, Lebailly P, Mohammed-Brahim B, et al. Neurodegenerative diseases and exposure to pesticides in the elderly. Am J Epidemiol 2003;157(5):409–414.](http://paperpile.com/b/9ckeIm/FyWx)

e28.	[Duzcan F, Zencir M, Ozdemir F, et al. Familial influence on parkinsonism in a rural area of Turkey (Kizilcaboluk-Denizli): a community-based case-control study. Mov Disord 2003;18(7):799–804.](http://paperpile.com/b/9ckeIm/y55O)

e29.	[Elbaz A, Levecque C, Clavel J, et al. S18Y polymorphism in the UCH-L1 gene and Parkinson’s disease: evidence for an age-dependent relationship. Mov Disord 2003;18(2):130–137.](http://paperpile.com/b/9ckeIm/H9Rq)

e30.	[Pals P, Van Everbroeck B, Grubben B, et al. Case-control study of environmental risk factors for Parkinson’s disease in Belgium. Eur J Epidemiol 2003;18(12):1133–1142.](http://paperpile.com/b/9ckeIm/ziCT)

e31.	[Elbaz A, Levecque C, Clavel J, et al. CYP2D6 polymorphism, pesticide exposure, and Parkinson’s disease. Ann Neurol 2004;55(3):430–434.](http://paperpile.com/b/9ckeIm/yV5v)

e32.	[Nuti A, Ceravolo R, Dell’Agnello G, et al. Environmental factors and Parkinson’s disease: a case-control study in the Tuscany region of Italy. Parkinsonism Relat Disord 2004;10(8):481–485.](http://paperpile.com/b/9ckeIm/IWUV)

e33.	[Gorell JM, Peterson EL, Rybicki BA, Johnson CC. Multiple risk factors for Parkinson’s disease. J Neurol Sci 2004;217(2):169–174.](http://paperpile.com/b/9ckeIm/MBFu)

e34.	[Koldkjaer OG, Wermuth L, Bjerregaard P. Parkinson’s disease among Inuit in Greenland: organochlorines as risk factors. Int J Circumpolar Health 2004;63 Suppl 2:366–368.](http://paperpile.com/b/9ckeIm/hYTL)

e35.	[Wright JM, Keller-Byrne J. Environmental determinants of Parkinson’s disease. Arch Environ Occup Health 2005;60(1):32–38.](http://paperpile.com/b/9ckeIm/kS2S)

e36.	[Fong C-S, Cheng C-W, Wu R-M. Pesticides exposure and genetic polymorphism of paraoxonase in the susceptibility of Parkinson’s disease. Acta Neurol Taiwan 2005;14(2):55–60.](http://paperpile.com/b/9ckeIm/6jKC)

e37.	[Galanaud J-P, Elbaz A, Clavel J, et al. Cigarette smoking and Parkinson’s disease: a case-control study in a population characterized by a high prevalence of pesticide exposure. Mov Disord 2005;20(2):181–189.](http://paperpile.com/b/9ckeIm/hTBD)

e38.	[Firestone JA, Smith-Weller T, Franklin G, et al. Pesticides and risk of Parkinson disease: a population-based case-control study. Arch Neurol 2005;62(1):91–95.](http://paperpile.com/b/9ckeIm/wjIM)

e39.	[Gartner CE, Battistutta D, Dunne MP, et al. Test-retest repeatability of self-reported environmental exposures in Parkinson’s disease cases and healthy controls. Parkinsonism Relat Disord 2005;11(5):287–295.](http://paperpile.com/b/9ckeIm/namd)

e40.	[Frigerio R, Sanft KR, Grossardt BR, et al. Chemical exposures and Parkinson’s disease: a population-based case-control study. Mov. Disord. 2006;21(10):1688–1692.](http://paperpile.com/b/9ckeIm/Dglf)

e41.	[Kelada SNP, Checkoway H, Kardia SLR, et al. 5’ and 3' region variability in the dopamine transporter gene (SLC6A3), pesticide exposure and Parkinson's disease risk: a hypothesis-generating study. Hum Mol Genet 2006;15(20):3055–3062.](http://paperpile.com/b/9ckeIm/LSX4)

e42.	[Ascherio A, Chen H, Weisskopf MG, et al. Pesticide exposure and risk for Parkinson’s disease. Ann Neurol 2006;60(2):197–203.](http://paperpile.com/b/9ckeIm/lAyV)

e43.	[Corder EH, Mellick GD. Parkinson’s disease in relation to pesticide exposure and nuclear encoded mitochondrial complex I gene variants. J Biomed Biotechnol 2006;2006(3):27601.](http://paperpile.com/b/9ckeIm/bhY6)

e44.	[Kamel F, Tanner C, Umbach D, et al. Pesticide exposure and self-reported Parkinson’s disease in the agricultural health study. Am J Epidemiol 2007;165(4):364–374.](http://paperpile.com/b/9ckeIm/ZZU8)

e45.	[Dick FD, De Palma G, Ahmadi A, et al. Environmental risk factors for Parkinson’s disease and parkinsonism: the Geoparkinson study. Occup Environ Med 2007;64(10):666–672.](http://paperpile.com/b/9ckeIm/fX43)

e46.	[Fong C-S, Wu R-M, Shieh J-C, et al. Pesticide exposure on southwestern Taiwanese with MnSOD and NQO1 polymorphisms is associated with increased risk of Parkinson’s disease. Clin Chim Acta 2007;378(1-2):136–141.](http://paperpile.com/b/9ckeIm/N40W)

e47.	[Dhillon AS, Tarbutton GL, Levin JL, et al. Pesticide/environmental exposures and Parkinson’s disease in East Texas. J Agromedicine 2008;13(1):37–48.](http://paperpile.com/b/9ckeIm/XAFE)

e48.	[Petersen MS, Halling J, Bech S, et al. Impact of dietary exposure to food contaminants on the risk of Parkinson’s disease. Neurotoxicology 2008;29(4):584–590.](http://paperpile.com/b/9ckeIm/hf9F)

e49.	[Brighina L, Frigerio R, Schneider NK, et al. Alpha-synuclein, pesticides, and Parkinson disease: a case-control study. Neurology 2008;70(16 Pt 2):1461–1469.](http://paperpile.com/b/9ckeIm/j8O0)

e50.	[Cho J-W, Jeon BS, Jeong D, et al. Association between parkinsonism and participation in agriculture in Korea. J Clin Neurol 2008;4(1):23–28.](http://paperpile.com/b/9ckeIm/knqJ)

e51.	[Hancock DB, Martin ER, Mayhew GM, et al. Pesticide exposure and risk of Parkinson’s disease: a family-based case-control study. BMC Neurol 2008;8:6.](http://paperpile.com/b/9ckeIm/I362)

e52.	[Gatto NM, Cockburn M, Bronstein J, et al. Well-water consumption and Parkinson’s disease in rural California. Environ Health Perspect 2009;117(12):1912–1918.](http://paperpile.com/b/9ckeIm/7uvC)

e53.	[Costello S, Cockburn M, Bronstein J, et al. Parkinson’s disease and residential exposure to maneb and paraquat from agricultural applications in the central valley of California. Am J Epidemiol 2009;169(8):919–926.](http://paperpile.com/b/9ckeIm/FW6r)

e54.	[Elbaz A, Clavel J, Rathouz PJ, et al. Professional exposure to pesticides and Parkinson disease. Ann Neurol 2009;66(4):494–504.](http://paperpile.com/b/9ckeIm/V9rV)

e55.	[Ritz BR, Manthripragada AD, Costello S, et al. Dopamine transporter genetic variants and pesticides in Parkinson’s disease. Environ Health Perspect 2009;117(6):964–969.](http://paperpile.com/b/9ckeIm/LcRE)

e56.	[Zschiedrich K, König IR, Brüggemann N, et al. MDR1 variants and risk of Parkinson disease. Association with pesticide exposure? J Neurol 2009;256(1):115–120.](http://paperpile.com/b/9ckeIm/vVOr)

e57.	[Tanner CM, Ross GW, Jewell SA, et al. Occupation and risk of parkinsonism: a multicenter case-control study. Arch Neurol 2009;66(9):1106–1113.](http://paperpile.com/b/9ckeIm/zGrs)

e58.	[Richardson JR, Shalat SL, Buckley B, et al. Elevated serum pesticide levels and risk of Parkinson disease. Arch Neurol 2009;66(7):870–875.](http://paperpile.com/b/9ckeIm/Ls7W)

e59.	[Firestone JA, Lundin JI, Powers KM, et al. Occupational factors and risk of Parkinson’s disease: A population-based case-control study. Am J Ind Med 2010;53(3):217–223.](http://paperpile.com/b/9ckeIm/nmGP)

e60.	[Kiyohara C, Miyake Y, Koyanagi M, et al. GST polymorphisms, interaction with smoking and pesticide use, and risk for Parkinson’s disease in a Japanese population. Parkinsonism Relat Disord 2010;16(7):447–452.](http://paperpile.com/b/9ckeIm/aVQ1)

e61.	[Skeie GO, Muller B, Haugarvoll K, et al. Differential effect of environmental risk factors on postural instability gait difficulties and tremor dominant Parkinson’s disease. Mov Disord 2010;25(12):1847–1852.](http://paperpile.com/b/9ckeIm/qaR4)

e62.	[Weisskopf MG, Knekt P, O’Reilly EJ, et al. Persistent organochlorine pesticides in serum and risk of Parkinson disease. Neurology 2010;74(13):1055–1061.](http://paperpile.com/b/9ckeIm/v9OH)

e63.	[Dutheil F, Beaune P, Tzourio C, et al. Interaction between ABCB1 and professional exposure to organochlorine insecticides in Parkinson disease. Arch Neurol 2010;67(6):739–745.](http://paperpile.com/b/9ckeIm/5HN1)

e64.	[Vlajinac HD, Sipetic SB, Maksimovic JM, et al. Environmental factors and Parkinson’s disease: a case-control study in Belgrade, Serbia. Int J Neurosci 2010;120(5):361–367.](http://paperpile.com/b/9ckeIm/3U5S)

e65.	[Sanyal J, Chakraborty DP, Sarkar B, et al. Environmental and familial risk factors of Parkinsons disease: case-control study. Can J Neurol Sci 2010;37(5):637–642.](http://paperpile.com/b/9ckeIm/tkno)

e66.	[Pereira D, Garrett C. [Risk factors for Parkinson disease: an epidemiologic study]. Acta Med Port 2010;23(1):15–24.](http://paperpile.com/b/9ckeIm/uDI8)

e67.	[Manthripragada AD, Costello S, Cockburn MG, et al. Paraoxonase 1, agricultural organophosphate exposure, and Parkinson disease. Epidemiology 2010;21(1):87–94.](http://paperpile.com/b/9ckeIm/Cz7q)

e68.	[Gatto NM, Rhodes SL, Manthripragada AD, et al. α-Synuclein gene may interact with environmental factors in increasing risk of Parkinson’s disease. Neuroepidemiology 2010;35(3):191–195.](http://paperpile.com/b/9ckeIm/yJMd)

e69.	[Wang A, Costello S, Cockburn M, et al. Parkinson’s disease risk from ambient exposure to pesticides. Eur J Epidemiol 2011;26(7):547–555.](http://paperpile.com/b/9ckeIm/aF1H)

e70.	[Rugbjerg K, Harris MA, Shen H, et al. Pesticide exposure and risk of Parkinson’s disease--a population-based case-control study evaluating the potential for recall bias. Scand J Work Environ Health 2011;37(5):427–436.](http://paperpile.com/b/9ckeIm/4Lrk)

e71.	[Richardson JR, Roy A, Shalat SL, et al. β-Hexachlorocyclohexane levels in serum and risk of Parkinson’s disease. Neurotoxicology 2011;32(5):640–645.](http://paperpile.com/b/9ckeIm/rJml)

e72.	[Das K, Ghosh M, Nag C, et al. Role of familial, environmental and occupational factors in the development of Parkinson’s disease. Neurodegener Dis 2011;8(5):345–351.](http://paperpile.com/b/9ckeIm/XD3s)

e73.	[Lin C-H, Wu R-M, Tai C-H, et al. Lrrk2 S1647T and BDNF V66M interact with environmental factors to increase risk of Parkinson’s disease. Parkinsonism Relat Disord 2011;17(2):84–88.](http://paperpile.com/b/9ckeIm/FA7R)

e74.	[Tanaka K, Miyake Y, Fukushima W, et al. Occupational risk factors for Parkinson’s disease: a case-control study in Japan. BMC Neurol 2011;11:83.](http://paperpile.com/b/9ckeIm/fOq0)

e75.	[Tanner CM, Kamel F, Ross GW, et al. Rotenone, paraquat, and Parkinson’s disease. Environ Health Perspect 2011;119(6):866–872.](http://paperpile.com/b/9ckeIm/5cZe)

e76.	[Infante J, Sierra M, Sánchez-Juan P, et al. Interaction between heme oxygenase-1 genotypes and exposure to pesticides in Parkinson’s disease. Mov Disord 2011;26(5):916–917.](http://paperpile.com/b/9ckeIm/UFUM)

e77.	[Goldman SM, Kamel F, Ross GW, et al. Genetic modification of the association of paraquat and Parkinson’s disease. Mov Disord 2012;27(13):1652–1658.](http://paperpile.com/b/9ckeIm/ggQR)

e78.	[Lee P-C, Bordelon Y, Bronstein J, Ritz B. Traumatic brain injury, paraquat exposure, and their relationship to Parkinson disease. Neurology 2012;79(20):2061–2066.](http://paperpile.com/b/9ckeIm/9AWw)

e79.	[Kiyohara C, Miyake Y, Koyanagi M, et al. MDR1 C3435T polymorphism and interaction with environmental factors in risk of Parkinson’s disease: a case-control study in Japan. Drug Metab Pharmacokinet 2013;28(2):138–143.](http://paperpile.com/b/9ckeIm/Gg6L)

e80.	[Lee P-C, Rhodes SL, Sinsheimer JS, et al. Functional paraoxonase 1 variants modify the risk of Parkinson’s disease due to organophosphate exposure. Environ Int 2013;56:42–47.](http://paperpile.com/b/9ckeIm/IKua)

e81.	[Pinhel MA de S, Sado CL, Longo GDS, et al. Nullity of GSTT1/GSTM1 related to pesticides is associated with Parkinson’s disease. Arq Neuropsiquiatr 2013;71(8):527–532.](http://paperpile.com/b/9ckeIm/Q2Mf)

e82.	[Narayan S, Liew Z, Paul K, et al. Household organophosphorus pesticide use and Parkinson’s disease. Int J Epidemiol 2013;42(5):1476–1485.](http://paperpile.com/b/9ckeIm/6Af2)

e83.	[Longo GS, Pinhel MS, Sado CL, et al. Exposure to pesticides and heterozygote genotype of GSTP1-Alw26I are associated to Parkinson’s disease. Arq Neuropsiquiatr 2013;71(7):446–452.](http://paperpile.com/b/9ckeIm/keoG)

e84.	[Chhillar N, Singh NK, Banerjee BD, et al. Organochlorine pesticide levels and risk of Parkinson’s disease in north Indian population. ISRN Neurol 2013;2013:371034.](http://paperpile.com/b/9ckeIm/eE79)

e85.	[Kamel F, Goldman SM, Umbach DM, et al. Dietary fat intake, pesticide use, and Parkinson’s disease. Parkinsonism Relat Disord 2014;20(1):82–87.](http://paperpile.com/b/9ckeIm/pHoP)

e86.	[van der Mark M, Vermeulen R, Nijssen PCG, et al. Occupational exposure to pesticides and endotoxin and Parkinson disease in the Netherlands. Occup Environ Med 2014;71(11):757–764.](http://paperpile.com/b/9ckeIm/myl4)

e87.	[Fitzmaurice AG, Rhodes SL, Cockburn M, et al. Aldehyde dehydrogenase variation enhances effect of pesticides associated with Parkinson disease. Neurology 2014;82(5):419–426.](http://paperpile.com/b/9ckeIm/1P1X)

e88.	[Wang A, Cockburn M, Ly TT, et al. The association between ambient exposure to organophosphates and Parkinson’s disease risk. Occup Environ Med 2014;71(4):275–281.](http://paperpile.com/b/9ckeIm/Ido0)

e89.	[Liew Z, Wang A, Bronstein J, Ritz B. Job exposure matrix (JEM)-derived estimates of lifetime occupational pesticide exposure and the risk of Parkinson’s disease. Arch Environ Occup Health 2014;69(4):241–251.](http://paperpile.com/b/9ckeIm/gNzz)

e90.	[Furlong M, Tanner CM, Goldman SM, et al. Protective glove use and hygiene habits modify the associations of specific pesticides with Parkinson’s disease. Environ Int 2015;75:144–150.](http://paperpile.com/b/9ckeIm/nyaa)

e91.	[Narayan S, Sinsheimer JS, Paul KC, et al. Genetic variability in ABCB1, occupational pesticide exposure, and Parkinson’s disease. Environ Res 2015;143(Pt A):98–106.](http://paperpile.com/b/9ckeIm/fWeu)

e92.	[Moisan F, Spinosi J, Delabre L, et al. Association of Parkinson’s Disease and Its Subtypes with Agricultural Pesticide Exposures in Men: A Case-Control Study in France. Environ Health Perspect 2015;123(11):1123–1129.](http://paperpile.com/b/9ckeIm/IPG0)

e93.	[Yang XL, Luo Q, Song HX, et al. Related factors and prevalence of Parkinson’s disease among Uygur residents in Hetian, Xinjiang Uygur Autonomous Region. Genet Mol Res 2015;14(3):8539–8546.](http://paperpile.com/b/9ckeIm/wXya)

e94.	[Beghdadli B, Ghomari O, Hamimed MEA, et al. Maladie de Parkinson et facteurs de risque professionnels et environnementaux : enquête cas-témoins dans l’ouest algérien. Arch. Mal. Prof. L Environ. 2016;77(1):21–26.](http://paperpile.com/b/9ckeIm/c4QT)

e95.	[Paul KC, Sinsheimer JS, Rhodes SL, et al. Organophosphate Pesticide Exposures, Nitric Oxide Synthase Gene Variants, and Gene-Pesticide Interactions in a Case-Control Study of Parkinson’s Disease, California (USA). Environ Health Perspect 2016;124(5):570–577.](http://paperpile.com/b/9ckeIm/wd8j)

e96.	[Schirinzi T, Martella G, D’Elia A, et al. Outlining a Population “at Risk” of Parkinson’s Disease: Evidence from a Case-Control Study. Parkinsons Dis 2016;2016:9646057.](http://paperpile.com/b/9ckeIm/CYPU)

e97.	[Biernacka JM, Chung SJ, Armasu SM, et al. Genome-wide gene-environment interaction analysis of pesticide exposure and risk of Parkinson’s disease. Parkinsonism Relat Disord 2016;32:25–30.](http://paperpile.com/b/9ckeIm/0bVe)

e98.	[Chuang C-S, Su H-L, Lin C-L, Kao C-H. Risk of Parkinson disease after organophosphate or carbamate poisoning. Acta Neurol Scand 2017;136(2):129–137.](http://paperpile.com/b/9ckeIm/gIis)

e99.	[Sanders LH, Paul KC, Howlett EH, et al. Editor’s Highlight: Base Excision Repair Variants and Pesticide Exposure Increase Parkinson's Disease Risk. Toxicol Sci 2017;158(1):188–198.](http://paperpile.com/b/9ckeIm/YDtp)

e100.	[Brouwer M, Huss A, van der Mark M, et al. Environmental exposure to pesticides and the risk of Parkinson’s disease in the Netherlands. Environ Int 2017;107:100–110.](http://paperpile.com/b/9ckeIm/Pp2h)

e101.	[Narayan S, Liew Z, Bronstein JM, Ritz B. Occupational pesticide use and Parkinson’s disease in the Parkinson Environment Gene (PEG) study. Environ Int 2017;107:266–273.](http://paperpile.com/b/9ckeIm/3VkL)

e102.	[Pouchieu C, Piel C, Carles C, et al. Pesticide use in agriculture and Parkinson’s disease in the AGRICAN cohort study. Int J Epidemiol 2018;47(1):299–310.](http://paperpile.com/b/9ckeIm/YFAS)

e103.	[Rösler TW, Salama M, Shalash AS, et al. K-variant BCHE and pesticide exposure: Gene-environment interactions in a case-control study of Parkinson’s disease in Egypt. Sci Rep 2018;8(1):16525.](http://paperpile.com/b/9ckeIm/C0IE)

e104.	[Paul KC, Sinsheimer JS, Cockburn M, et al. NFE2L2, PPARGC1α, and pesticides and Parkinson’s disease risk and progression. Mech Ageing Dev 2018;173:1–8.](http://paperpile.com/b/9ckeIm/hTWC)

e105.	[Tufail M. Clinical Features and Risk Factors of Parkinson’s Disease in a Population of Khyber Pakhtunkhwa, Pakistan: A Case-Control Study. Neurodegener Dis 2019;19(5-6):211–217.](http://paperpile.com/b/9ckeIm/f8Pl)

e106.	[Goldman SM, Tanner CM. Rotenone and Parkinson’s Disease: Reduced Sensitivity in Females. Toxicol Sci 2019;170(2):562–563.](http://paperpile.com/b/9ckeIm/Mc5H)

e107.	[Angelopoulou E, Bozi M, Simitsi A-M, et al. The relationship between environmental factors and different Parkinson’s disease subtypes in Greece: Data analysis of the Hellenic Biobank of Parkinson's disease. Parkinsonism Relat Disord 2019;67:105–112.](http://paperpile.com/b/9ckeIm/9FFb)

e108.	[Ventura-Chávez R, Reyna-Gil AI, García S. Factores asociados a la enfermedad de Parkinson en pacientes de la Comarca Lagunera, México [Internet]. Revista Mexicana de Neurociencia 2019;20(4)Available from:](http://paperpile.com/b/9ckeIm/SYvT) <http://www.revmexneurociencia.com/frame_esp.php?id=48>

e109.	[Georgiou A, Demetriou CA, Christou YP, et al. Genetic and Environmental Factors Contributing to Parkinson’s Disease: A Case-Control Study in the Cypriot Population. Front Neurol 2019;10:1047.](http://paperpile.com/b/9ckeIm/YuoM)

e110.	Calvo-Trujillo M, Mendoza-Goez L, García-Espiñeira M, et al. Exposure to pesticides as a risk factor for Parkinson's disease: a case-control study in San Juan Nepomuceno town (Bolivar). Rev Toxicol 2019;36(2):142-147.

e111.	[Dardiotis E, Aloizou A-M, Sakalakis E, et al. Organochlorine pesticide levels in Greek patients with Parkinson’s disease. Toxicol Rep 2020;7:596–601.](http://paperpile.com/b/9ckeIm/lvgG)

e112.	[Silvestre GCSB, Ferreira MJM, Figueiredo SEFMR de, et al. Parkinson Disease and Occupational and Environmental Exposure to Pesticides in a Region of Intense Agribusiness Activity in Brazil: A Case-Control Study. J Occup Environ Med 2020;62(12):e732–e737.](http://paperpile.com/b/9ckeIm/Fmsh)

e113.	[Shrestha S, Parks CG, Umbach DM, et al. Pesticide use and incident Parkinson’s disease in a cohort of farmers and their spouses. Environ Res 2020;191:110186.](http://paperpile.com/b/9ckeIm/eANf)

e114.	[Belvisi D, Pellicciari R, Fabbrini A, et al. Risk factors of Parkinson disease: Simultaneous assessment, interactions, and etiologic subtypes. Neurology 2020;95(18):e2500–e2508.](http://paperpile.com/b/9ckeIm/WaeB)

e115.	[Poortvliet PC, Gluch A, Silburn PA, Mellick GD. The Queensland Parkinson’s Project: An Overview of 20 Years of Mortality from Parkinson's Disease. J Mov Disord 2021;14(1):34–41.](http://paperpile.com/b/9ckeIm/5NNL)

e116.	[Xu S, Yang X, Qian Y, et al. Analysis of serum levels of organochlorine pesticides and related factors in Parkinson’s disease. Neurotoxicology 2022;88:216–223.](http://paperpile.com/b/9ckeIm/Qewu)

e117.	[Shermon S, Goldfinger M, Morris A, et al. Effect of modifiable risk factors in Parkinson’s disease: A case-control study looking at common dietary factors, toxicants, and anti-inflammatory medications. Chronic Illn 2022;18(4):849–859.](http://paperpile.com/b/9ckeIm/WpZK)

e118.	[Belingheri M, Chiu Y-HM, Renzetti S, et al. Relationships of Nutritional Factors and Agrochemical Exposure with Parkinson’s Disease in the Province of Brescia, Italy [Internet]. Int J Environ Res Public Health 2022;19(6)Available from:](http://paperpile.com/b/9ckeIm/BbRG) <http://dx.doi.org/10.3390/ijerph19063309>

e119.	[Payami H, Cohen G, Murchison CF, et al. Population fraction of Parkinson’s disease attributable to preventable risk factors. NPJ Parkinsons Dis 2023;9(1):159.](http://paperpile.com/b/9ckeIm/iXPb)

e120.	[Moura DD, Borges V, Ferraz HB, et al. History of high household pesticide use and Parkinson’s disease in Brazil. Parkinsonism Relat. Disord. 2023;113:105493.](http://paperpile.com/b/9ckeIm/La10)

e121.	[Paul KC, Cockburn M, Gong Y, et al. Agricultural paraquat dichloride use and Parkinson’s disease in California's Central Valley [Internet]. Int J Epidemiol 2024;53(1)Available from:](http://paperpile.com/b/9ckeIm/1uHl) <http://dx.doi.org/10.1093/ije/dyae004>

e122.	[Kumar B, Saluja A, Juneja A, et al. Role of PON1 L55M Gene Polymorphism in Parkinson’s Disease among North Indian Population. Neurol India 2024;72(2):364–367.](http://paperpile.com/b/9ckeIm/rV17)

e123.	[Reynoso A, Torricelli R, Jacobs BM, et al. Gene-Environment Interactions for Parkinson’s Disease. Ann Neurol 2024;95(4):677–687.](http://paperpile.com/b/9ckeIm/6B0E)

e124.	[Schaffner SL, Casazza W, Artaud F, et al. Genetic variation and pesticide exposure influence blood DNA methylation signatures in females with early-stage Parkinson’s disease. NPJ Parkinsons Dis 2024;10(1):98.](http://paperpile.com/b/9ckeIm/TC12)
