# Supplementary material for: Revisiting the Association of Pesticide Exposure and Parkinson's Disease: Systematic Review and Meta‐Analysis
Source: Environ Toxicol. 2026 Jan 21;41(10):875–88. doi: 10.1002/tox.70041 (PMC13537366; doi:10.1002/tox.70041)
Supplement: Supplementary file 2 [file TOX-41-875-s003.docx]

**Supplementary Table S5.** Meta-analysis of the association among pesticide exposure and Parkinson's disease according to study quality by the Newcastle-Ottawa Scale.

| **Exposure** | **Pesticide class** | **K** | **Random-effects model** | | | **Heterogeneity** |
| --- | --- | --- | --- | --- | --- | --- |
|  |  |  | **OR** | **95% CI** | **p-value** | **I^2^** |
| Any type | Any pesticides | 44 | **1.80** | **1.51-2.14** | **< 0.0001** | 83.45 |
|  | NOS < 6 | 39 | **1.73** | **1.44-2.08** | **< 0.0001** | 83.15 |
|  | NOS ≥ 6 | 5 | **2.39** | **1.35-4.22** | **0.0028** | 81.56 |
|  | Insecticides | 12 | 1.43 | 1.00-2.05 | 0.0524 | 87.75 |
|  | NOS < 6 | 10 | 1.41 | 0.90-2.21 | 0.1356 | 90.23 |
|  | NOS ≥ 6 | 2 | **1.65** | **1.26-2.17** | **0.0003** | 0.00 |
|  | Herbicides | 11 | **1.58** | **1.26-1.97** | **< 0.0001** | 72.49 |
|  | NOS < 6 | 10 | **1.60** | **1.23-2.08** | **0.0004** | 77.52 |
|  | NOS ≥ 6 | 1 | - | - | - | 0.00 |
|  | Fungicides | 4 | 1.45 | 0.70-2.99 | 0.3166 | 78.56 |
|  | NOS < 6 | 4 | 1.45 | 0.70-2.99 | 0.3166 | 78.56 |
|  | NOS ≥ 6 | 0 | - | - | - | - |
| Occupational | Any pesticides | 40 | **1.46** | **1.27-1.68** | **< 0.0001** | 71.37 |
|  | NOS < 6 | 31 | **1.50** | **1.26-1.78** | **< 0.0001** | 77.30 |
|  | NOS ≥ 6 | 9 | **1.36** | **1.08-1.71** | **0.0094** | 38.78 |
|  | Insecticides | 20 | **1.46** | **1.13-1.88** | **0.0033** | 75.36 |
|  | NOS < 6 | 14 | **1.47** | **1.00-2.17** | **0.0473** | 83.89 |
|  | NOS ≥ 6 | 6 | **1.48** | **1.20-1.82** | **0.0002** | 0.00 |
|  | Herbicides | 21 | **1.27** | **1.08-1.50** | **0.0045** | 59.37 |
|  | NOS < 6 | 14 | 1.18 | 0.97-1.43 | 0.0966 | 56.47 |
|  | NOS ≥ 6 | 7 | **1.47** | **1.08-1.99** | **0.0136** | 54.90 |
|  | Fungicides | 15 | 1.25 | 0.99-1.60 | 0.0656 | 64.71 |
|  | NOS < 6 | 12 | 1.14 | 0.90-1.44 | 0.2761 | 49.96 |
|  | NOS ≥ 6 | 3 | **1.70** | **1.01-2.87** | **0.0463** | 58.60 |
| Household | Any pesticides | 23 | **1.21** | **1.06-1.39** | **0.0058** | 75.15 |
|  | NOS < 6 | 18 | **1.24** | **1.04-1.47** | **0.0151** | 81.93 |
|  | NOS ≥ 6 | 5 | 1.10 | 0.91-1.32 | 0.3477 | 0.00 |
|  | Insecticides | 8 | 1.29 | 0.98-1.70 | 0.0699 | 72.29 |
|  | NOS < 6 | 7 | **1.40** | **1.07-1.83** | **0.0132** | 63.20 |
|  | NOS ≥ 6 | 1 | - | - | - | 0.00 |
| Any type | Paraquat (herbicide) | 6 | **1.34** | **1.13-1.60** | **0.0007** | 49.92 |
|  | NOS < 6 | 6 | **1.34** | **1.13-1.60** | **0.0007** | 49.92 |
|  | NOS ≥ 6 | 0 | **-** | **-** | **-** | - |
| Occupational | Paraquat (herbicide) | 8 | 1.21 | 0.96-1.53 | 0.1061 | 72.31 |
|  | NOS < 6 | 5 | 1.10 | 0.83-1.44 | 0.5158 | 71.94 |
|  | NOS ≥ 6 | 3 | **1.53** | **1.11-2.12** | **0.0101** | 0.00 |

Abbreviations: CI, confidence interval; I^2^, Higgin's & Thompson's I^2^ statistic; K, total number of studies on pesticide exposure; NOS, Newcastle-Ottawa Scale; OR, odds ratio.

Bold text indicates statistically significant associations.
