# Supplementary material for: Revisiting the Association of Pesticide Exposure and Parkinson's Disease: Systematic Review and Meta‐Analysis
Source: Environ Toxicol. 2026 Jan 21;41(10):875–88. doi: 10.1002/tox.70041 (PMC13537366; doi:10.1002/tox.70041)
Supplement: Supplementary file 3 [file TOX-41-875-s004.docx]

**Supplementary Table S2.** Number of participants from selected studies per global subregion.

| **Global subregion** | **n Studies** | **n People with PD** | **n Male People with PD** | **n Female People with PD** | **n Controls** | **n Male Controls** | **n Female Controls** |
| --- | --- | --- | --- | --- | --- | --- | --- |
| North America | 55 | 15,210 | 8,180* | 5,190* | 314,302 | 53,942* | 36,498* |
| Europe | 35 | 9,624 | 5,368 | 3,969 | 160,897 | 87,421 | 71,973 |
| Asia | 19 | 3,852 | 2,128 | 1,576 | 50,653 | 34,139 | 15,821 |
| Central and South America | 6 | 1,163 | 493* | 416* | 1,119 | 401* | 549* |
| Oceania | 6 | 2,086 | 1,117* | 634* | 2,004 | 924* | 759* |
| Africa | 2 | 737 | 427 | 310 | 767 | 437 | 330 |
| Total | 124**^§^** | 32,825 | 17,785* | 12,176* | 529,897 | 177,419* | 126,019* |

Abbreviations: n, total number of participants (counts) included in studies; PD, Parkinson’s disease.

§ One study [e123] could not be categorized by subregion due to a global recruitment strategy (genetic testing company consumers)

* Studies that did not describe the number of male and female participants: North America – 7 studies; Central and South America – 1 study; Oceania – 1 study
