# Supplementary material for: Revisiting the Association of Pesticide Exposure and Parkinson's Disease: Systematic Review and Meta‐Analysis
Source: Environ Toxicol. 2026 Jan 21;41(10):875–88. doi: 10.1002/tox.70041 (PMC13537366; doi:10.1002/tox.70041)
Supplement: Supplementary file 4 [file TOX-41-875-s006.docx]

**Supplementary Table S3.** Influence of methodological variables as sources of heterogeneity among studies included in meta-analyses showing the association between pesticide exposure and risk of developing Parkinson’s disease

| **Exposure** | **Variable** | **OR (95% CI)** | **Heterogeneity**  **I^2^** | **Univariate metaregression** | |
| --- | --- | --- | --- | --- | --- |
|  |  |  |  | **β coefficient** | **p-value** |
| **Any type** | |  |  |  | |
| Any pesticides |  | **1.80 (1.51-2.14)** | **83.45** |  |  |
|  | Study quality |  |  | 0.0453 | 0.4283 |
|  | Rural/urban setting |  |  | **-0.8782** | **0.0059** |
|  | Number of participants |  |  | 0.0000 | 0.8686 |
|  | Global subregion |  |  | -0.0441 | 0.4455 |
|  | Method of evaluation |  |  | 0.0731 | 0.3859 |
|  | Publication year |  |  | 0.0271 | 0.6042 |
| Insecticides |  | 1.43 (1.00-2.05) | 87.75 |  |  |
|  | Study quality |  |  | 0.0568 | 0.7044 |
|  | Rural/urban setting |  |  | 0.0792 | 0.8242 |
|  | Number of participants |  |  | -0.0005 | 0.2529 |
|  | Global subregion |  |  | -0.1995 | 0.1093 |
|  | Method of evaluation |  |  | -0.0651 | 0.6847 |
|  | Publication year |  |  | -0.0021 | 0.9765 |
| Herbicides |  | **1.58 (1.26-1.97)** | **72.49** |  |  |
|  | Study quality |  |  | -0.0642 | 0.3985 |
|  | Rural/urban setting |  |  | 0.3372 | 0.0709 |
|  | Number of participants |  |  | -0.0002 | 0.6056 |
|  | Global subregion |  |  | **0.1890** | **0.0047** |
|  | Method of evaluation |  |  | -0.3372 | 0.0709 |
|  | Publication year |  |  | 0.0086 | 0.9328 |
| Fungicides |  | 1.45 (0.70-2.99) | 78.56 |  |  |
|  | Study quality |  |  | **-0.5014** | **0.0004** |
|  | Rural/urban setting |  |  | -0.1916 | 0.7774 |
|  | Number of participants |  |  | **-0.0028** | **<0.0001** |
|  | Global subregion |  |  | **-0.4813** | **0.0007** |
|  | Method of evaluation |  |  | 0.1916 | 0.7774 |
|  | Publication year |  |  | **-1.4160** | **0.0004** |
| **Occupational** | |  |  |  | |
| Any pesticides |  | **1.46 (1.27-1.68)** | **71.37** |  |  |
|  | Study quality |  |  | -0.0016 | 0.9719 |
|  | Rural/urban setting |  |  | -0.0182 | 0.8980 |
|  | Number of participants |  |  | -0.0000 | 0.3311 |
|  | Global subregion |  |  | -0.0475 | 0.4765 |
|  | Method of evaluation |  |  | -0.0180 | 0.8304 |
|  | Publication year |  |  | 0.0068 | 0.7875 |
| Insecticides |  | **1.46 (1.13-1.88)** | **75.36** |  |  |
|  | Study quality |  |  | 0.0038 | 0.9576 |
|  | Rural/urban setting |  |  | 0.0599 | 0.8138 |
|  | Number of participants |  |  | **-0.0000** | **0.0075** |
|  | Global subregion |  |  | 0.1563 | 0.1869 |
|  | Method of evaluation |  |  | 0.4342 | 0.1241 |
|  | Publication year |  |  | **0.2785** | **0.0265** |
| Herbicides |  | **1.27 (1.08-1.50)** | **59.37** |  |  |
|  | Study quality |  |  | 0.2534 | 0.4686 |
|  | Rural/urban setting |  |  | 0.1057 | 0.5449 |
|  | Number of participants |  |  | **-0.0000** | **<0.0001** |
|  | Global subregion |  |  | 0.1311 | 0.0740 |
|  | Method of evaluation |  |  | 0.0500 | 0.0854 |
|  | Publication year |  |  | -0.1088 | 0.1993 |
| Fungicides |  | 1.25 (0.99-1.60) | 64.71 |  |  |
|  | Study quality |  |  | 0.0503 | 0.4014 |
|  | Rural/urban setting |  |  | -0.1096 | 0.6416 |
|  | Number of participants |  |  | -0.0000 | 0.2009 |
|  | Global subregion |  |  | -0.0036 | 0.9750 |
|  | Method of evaluation |  |  | 0.1531 | 0.6197 |
|  | Publication year |  |  | 0.1803 | 0.2054 |
| **Household** | |  |  |  |  |
| Any pesticides |  | **1.21 (1.06-1.39)** | **75.15** |  |  |
|  | Study quality |  |  | -0.0424 | 0.2518 |
|  | Rural/urban setting |  |  | -0.1243 | 0.3626 |
|  | Number of participants |  |  | -0.0000 | 0.9202 |
|  | Global subregion |  |  | 0.0586 | 0.3071 |
|  | Method of evaluation |  |  | 0.0120 | 0.9505 |
|  | Publication year |  |  | 0.0596 | 0.4794 |
| Insecticides |  | 1.29 (0.98-1.70) | 72.29 |  |  |
|  | Study quality |  |  | -0.1009 | 0.2747 |
|  | Rural/urban setting |  |  | -0.2698 | 0.2506 |
|  | Number of participants |  |  | 0.0004 | 0.4282 |
|  | Global subregion |  |  | -0.6231 | 0.0829 |
|  | Method of evaluation |  |  | 0.1806 | 0.5504 |
|  | Publication year |  |  | 0.0056 | 0.9828 |
| **Paraquat** | |  |  |  |  |
| Any type of exposure |  | **1.34 (1.13-1.60)** | **49.92** |  |  |
|  | Study quality |  |  | **-0.1723** | **0.0077** |
|  | Rural/urban setting |  |  | -0.0021 | 0.9940 |
|  | Number of participants |  |  | 0.0000 | 0.8319 |
|  | Global subregion |  |  | **0.2233** | **0.0027** |
|  | Method of evaluation |  |  | 0.0021 | 0.9940 |
|  | Publication year |  |  | -0.0605 | 0.6230 |
| Occupational |  | 1.21 (0.96-1.53) | 72.31 |  |  |
|  | Study quality |  |  | 0.1054 | 0.0645 |
|  | Rural/urban setting |  |  | 0.0265 | 0.9452 |
|  | Number of participants |  |  | **-0.0000** | **<0.0001** |
|  | Global subregion |  |  | **0.2561** | **<0.0001** |
|  | Method of evaluation |  |  | -0.0060 | 0.9804 |
|  | Publication year |  |  | -0.0857 | 0.6350 |

Abbreviations: CI, confidence interval; I^2^, Higgin's & Thompson's I^2^ statistic; NA, not available; OR, odds ratio.

Bold text indicates the statistically significant influence of methodological variables on meta-analyses.
