# Supplementary material for: Revisiting the Association of Pesticide Exposure and Parkinson's Disease: Systematic Review and Meta‐Analysis
Source: Environ Toxicol. 2026 Jan 21;41(10):875–88. doi: 10.1002/tox.70041 (PMC13537366; doi:10.1002/tox.70041)
Supplement: Supplementary file 5 [file TOX-41-875-s007.docx]

**Supplementary Table S4.** Meta-analysis of the association among pesticide exposure and Parkinson's disease after excluding studies from leading research groups.

| **Exposure** | **Pesticide class** | **K** | **n** | **Random-effects model** | | | **Heterogeneity** |
| --- | --- | --- | --- | --- | --- | --- | --- |
|  |  |  |  | **OR** | **95% CI** | **p-value** | **I^2^** |
| **Excluding studies from Ritz group (USA, n = 18)** | | | | | | | |
| Any type | Any pesticides | 42 | 170,739 | **1.73** | **1.44-2.06** | **< 0.0001** | 83.04 |
|  | Insecticides | 8 | 4,589 | 1.43 | 0.93-2.20 | 0.1033 | 87.13 |
|  | Herbicides | 8 | 4,582 | **1.74** | **1.20-2.53** | **0.0036** | 80.02 |
|  | Fungicides | 4 | 2,649 | 1.45 | 0.70-2.99 | 0.3166 | 78.56 |
| Occupational | Any pesticides | 33 | 98,797 | **1.48** | **1.22-1.79** | **< 0.0001** | 77.03 |
|  | Insecticides | 17 | 235,520 | **1.34** | **1.02-1.77** | **0.0340** | 71.49 |
|  | Herbicides | 18 | 236,068 | **1.28** | **1.04-1.56** | 0.**0172** | 60.50 |
|  | Fungicides | 12 | 233,935 | 1.16 | 0.87-1.55 | 0.3221 | 66.14 |
| Household | Any pesticides | 17 | 249,615 | 1.08 | 0.95-1.23 | 0.2542 | 53.14 |
|  | Insecticides | 3 | 1,173 | 0.83 | 0.64-1.08 | 0.1594 | 0.00 |
| Any type | Paraquat (herbicide) | 2 | 1,319 | 1.07 | 0.76-1.49 | 0.7127 | 43.73 |
| Occupational | Paraquat (herbicide) | 6 | 231,071 | 1.25 | 0.91-1.71 | 0.1619 | 64.32 |
| **Excluding studies from Elbaz group (France, n = 7)** | | | | | | | |
| Any type | Any pesticides | *No studies were excluded from original analysis* | | | | | |
|  | Insecticides | 11 | 7,199 | 1.40 | 0.94-2.08 | 0.0961 | 89.33 |
|  | Herbicides | *No studies were excluded from original analysis* | | | | | |
|  | Fungicides | *No studies were excluded from original analysis* | | | | | |
| Occupational | Any pesticides | 34 | 101,099 | **1.54** | **1.29-1.82** | **< 0.0001** | 75.98 |
|  | Insecticides | 18 | 236,495 | **1.49** | **1.11-2.01** | **0.0079** | 77.88 |
|  | Herbicides | 19 | 238,245 | **1.32** | **1.10-1.58** | **0.0034** | 61.79 |
|  | Fungicides | 13 | 235,167 | 1.12 | 0.89-1.41 | 0.3296 | 47.41 |
| Household | Any pesticides | 16 | 251,678 | **1.24** | **1.02-1.51** | **0.0312** | 85.77 |
|  | Insecticides | *No studies were excluded from original analysis* | | | | | |
| Any type | Paraquat (herbicide) | *No studies were excluded from original analysis* | | | | | |
| Occupational | Paraquat (herbicide) | *No studies were excluded from original analysis* | | | | | |

Abbreviations: b, Egger’s test intercept; CI, confidence interval; I^2^, Higgin's & Thompson's I^2^ statistic; K, total number of studies on a pesticide exposure; n, total number of participants (counts) included in studies on a pesticide exposure; OR, odds ratio.

Bold text indicates statistically significant associations.
