# Supplementary material for: Revisiting the Association of Pesticide Exposure and Parkinson's Disease: Systematic Review and Meta‐Analysis
Source: Environ Toxicol. 2026 Jan 21;41(10):875–88. doi: 10.1002/tox.70041 (PMC13537366; doi:10.1002/tox.70041)
Supplement: Supplementary file 6 [file TOX-41-875-s008.docx]

**Supplementary Table S1.** Characteristics of studies selected in a meta-analysis for the association of pesticide exposure and Parkinson's disease

| **Author and Year** | **Population country** | **Global subregion** | **Sample size (PD/control)** | **Male sex (%) (PD/control)** | **AAE (PD/control)** | **Newcastle-Ottawa scale** | **Rural area** | **Type of exposure** | **Pesticide class analyzed** | **Any positive association between pesticide exposure and PD*** |  |
| --- | --- | --- | --- | --- | --- | --- | --- | --- | --- | --- | --- |
|  |  |  |  |  |  |  |  |  |  |  |  |
| Ho et al., 1989 [e1] | Hong Kong | Eastern Asia | 54/647 | 20/27 | ND/ND | 1/9 | Yes | Any exposure | Any class | Positive |  |
| Koller et al., 1990 [e2] | USA | Northern America | 150/150 | 59/59 | 66/66 | 5/9 | Yes | Any exposure | Any class | Negative |  |
| Golbe et al., 1990 [e3] | USA | Northern America | 106/106 | 67/67 | ND/ND | 3/9 | Yes | Any exposure | Any class | Positive |  |
| Wong et al., 1991 [e4] | USA | Northern America | 38/38 | 58/58 | 70/70 | 5/9 | Yes | Any exposure | Any class | ND |  |
| Stern et al., 1991 [e5] | USA | Northern America | 149/149 | 60/ND | ND/ND | 5/9 | No | Any exposure | Insecticide, herbicide | Negative |  |
|  |  |  |  |  |  |  |  |  |  |  |  |
| Semchuk et al., 1992 [e6] | Canada | Northern America | 130/260 | 58/58 | 68/68 | 4/9 | No | Occupational | Insecticide, herbicide, fungicide | Negative |  |
| Jiménez-Jiménez et al., 1992 [e7] | Spain | Southern Europe | 128/256 | 52/52 | 66/64 | 1/9 | No | Any exposure | Any class | Negative |  |
| Butterfield et al., 1993 [e8] | USA | Northern America | 63/68 | 63/51 | 49/50 | 5/9 | No | Any exposure | Insecticide, herbicide | Positive |  |
| Wang et al., 1994 [e9] | Canada | Northern America | 43/109 | ND/ND | ND/ND | 4/9 | No | Occupational | Insecticide, herbicide, fungicide | Negative |  |
| Hertzman et al., 1994 [e10] | Canada | Northern America | 127/245 | 56/57 | 70/67 | 5/9 | Yes | Occupational | Insecticide, herbicide, fungicide | Negative |  |
| Morano et al., 1994 [e11] | Spain | Southern Europe | 74/148 | 45/45 | 68/67 | 4/9 | No | Any exposure | Any class | ND |  |
| Chaturvedi et al., 1995 [e12] | Canada | Northern America | 87/2,070 | ND/ND | ND/ND | 1/9 | No | Any exposure | Any class | Positive |  |
| Semchuk et al., 1995 [e13] | Canada | Northern America | 130/260 | 58/58 | 68/68 | 5/9 | No | Occupational | Any class | Positive |  |
| Seidler et al., 1996 [e14] | Germany | Northern Europe | 380/755 | 66/ND | 56/56 | 6/9 | No | Any exposure | Insecticide, herbicide | Positive |  |
| Liou et al., 1997 [e15] | Taiwan | Eastern Asia | 120/240 | 54/54 | 63/63 | 6/9 | No | Any exposure | Herbicide | Positive |  |
| McCann et al., 1998 [e16] | Australia | Oceania | 224/310 | 58/62 | 70/68 | 4/9 | Yes | Any exposure | Any class | Positive |  |
| Gorell et al., 1998 [e17] | USA | Northem America | 144/464 | ND/ND | ND/ND | 4/9 | No | Any exposure | Herbicide, insecticide, fungicide | Positive |  |
| Chan et al., 1998 [e18] | Hong Kong | Eastern Asia | 215/313 | 53/55 | ND/ND | 5/9 | No | Occupational | Any class | Positive |  |
| Menegon et al., 1998 [e19] | Australia | Oceania | 98/95 | 60/77 | 72/67 | 1/9 | No | Any exposure | Any class | Positive |  |
| Werneck et al., 1999 [e20] | Brazil | Southern America | 92/110 | 45/43 | 70/68 | 5/9 | No | Any exposure | Herbicide and insecticide | Positive |  |
| Fall et al., 1999 [e21] | Sweden | Northern Europe | 113/263 | ND/ND | 62/58 | 5/9 | No | Occupational | Insecticides | Negative |  |
| Kuopio t al., 1999 [e22] | Finland | Northern Europe | 123/246 | 51/51 | 68/69 | 6/9 | No | Occupational | Herbicides | Negative |  |
| Taylor et al., 2000 [e23] | Australia | Oceania | 92/122 | 68/78 | ND/ND | 3/9 | No | Any exposure | Any class | Positive |  |
| Herishanu et al., 2001 [e24} | Israel | Western Asia | 93/93 | ND/ND | ND/ND | 5/9 | No | Any exposure | Any class | Negative |  |
| Zorzon et al., 2002 [e25] | Italy | Southern Europe | 132/272 | 46/ND | 70/69 | 5/9 | No | Any exposure | Any class | Positive |  |
| Petrovitch et al., 2002 [e26] | USA | Northem America | 116/7870 | 100/100 | ND/ND | 4/9 | No | Any exposure | Any class | Positive |  |
| Baldi et al., 2003 [e27] | France | Western Europe | 84/252 | 52/52 | 75/75 | 6/9 | Yes | Occupational | Any class | Positive |  |
| Duzcan et al., 2003 [e28] | Turkey | Western Asia | 36/108 | 47/ND | ND/ND | 7/9 | Yes | Any exposure | Any class | Positive |  |
| Elbaz et al., 2003 [e29] | France | Western Europe | 209/488 | 57/59 | 69/61 | 4/9 | Yes | Any exposure | Any class | Negative |  |
| Pals et al., 2003 [e30] | Belgium | Western Europe | 423/205 | 61/34 | 67/63 | 2/9 | No | Any exposure | Any class | Negative |  |
| Elbaz et al., 2004 [e31] | France | Western Europe | 190/419 | 56/56 | 67/67 | 5/9 | Yes | Any exposure | Any class | Positive |  |
| Nuti et al., 2004 [e32] | Italy | Southern Europe | 190/190 | 56/56 | 63/62 | 4/9 | No | Any exposure | Any class | Negative |  |
| Gorell et al., 2004 [e33] | USA | Northern America | 144/464 | ND/ND | ND/ND | 4/9 | No | Occupational | Herbicide, insecticide, fungicide | Positive |  |
| Koldkjaer et al., 2004 [e34] | Greenland | Northern Europe | 31/122 | 65/47 | 69/61 | 2/9 | Yes | Any exposure | Insecticides | Positive |  |
| Wright et al., 2005 [e35] | USA | Northern America | 102/133 | 51/36 | 71/73 | 1/9 | No | Any exposure | Any class | Positive |  |
| Fong et al., 2005 [e36] | Taiwan | Eastern Asia | 125/162 | 45/44 | 69/68 | 3/9 | No | Any exposure | Any class | Positive |  |
| Galanaud et al., 2005 [e37] | France | Western Europe | 247/676 | 56/56 | 69/69 | 7/9 | Yes | Any exposure | Any class | Positive |  |
| Firestone et al., 2005 [e38] | USA | Northern America | 250/388 | 62/62 | 70/70 | 6/9 | No | Any exposure | Herbicide, insecticide, fungicide | Negative |  |
| Gartner et al., 2005 [e39] | Australia | Oceania | 32/29 | 59/59 | 64/66 | 4/9 | No | Any exposure | Herbicide, insecticide, fungicide | ND |  |
| Frigerio et al., 2006 [e40] | USA | Northern America | 149/129 | 60/ND | ND/ND | 7/9 | No | Occupational | Any class | Positive |  |
| Kelada et al., 2006 [e41] | USA | Northern America | 293/395 | 65/62 | 66/69 | 5/9 | No | Occupational | Any class | Positive |  |
| Ascherio et al., 2006 [e42} | USA | Northern America | 413/142,912 | ND/ND | ND/63 | 3/9 | No | Any exposure | Any class | Positive |  |
| Corder et al., 2006 [e43] | Australia | Oceania | 306/321 | ND/ND | ND/ND | 1/9 | No | Any exposure | Any class | ND |  |
| Kamel et al., 2007 [e44} | USA | Northern America | 83/79,557 | ND/ND | ND/ND | 4/9 | No | Occupational | Any class | Positive |  |
| Dick et al., 2007 [e45] | Scotland, Sweden, Italy, Romania, Malta | Northern, Eastern and Southern Europe | 767/1,989 | 56/53 | 69/69 | 4/9 | No | Any exposure | Any class | Positive |  |
| Fong et al., 2007 [e46] | Taiwan | Eastern Asia | 153/155 | 47/43 | 71/70 | 5/9 | No | Any exposure | Any class | Positive |  |
| Dhillon et al., 2008 [e47] | USA | Northern America | 100/84 | 60/56 | 73/71 | 3/9 | No | Any exposure | Herbicide, insecticide, fungicide | Positive |  |
| Petersen et al., 2008 [e48] | Faroe Islands | Northern Europe | 79/154 | 54/55 | 74/75 | 6/9 | No | Occupational | Any class | Positive |  |
| Brighina et al., 2008 [e49] | USA | Northern America | 833/833 | 64/57 | 67/66 | 4/9 | No | Any exposure | Herbicide, insecticide, fungicide | Positive |  |
| Cho et al., 2008 [e50] | South Korea | Eastern Asia | 235/77 | 50/49 | 67/64 | 2/9 | No | Occupational | Any class | Positive |  |
| Hancock et al., 2008 [e51] | USA | Northern America | 319/296 | 68/43 | 65/63 | 5/9 | No | Any exposure | Herbicide, insecticide | Positive |  |
| Gatto et al., 2009 [e52] | USA | Northern America | 368/341 | 56/52 | 69/67 | 4/9 | Yes | Household | Fungicide, insecticide, herbicide | Positive |  |
| Costello et al., 2009 [e53] | USA | Northern America | 368/341 | 56/52 | 68/67 | 4/9 | Yes | Household | Herbicide and fungicide | Positive |  |
| Elbaz et al., 2009 [e54] | France | Western Europe | 224/557 | 53/52 | 69/68 | 7/9 | Yes | Any exposure | Herbicide, insecticide, fungicide | Positive |  |
| Ritz et al., 2009 [e55] | USA | Northern America | 324/334 | 55/50 | ND/ND | 3/9 | Yes | Any exposure | Herbicide and fungicide | Positive |  |
| Zschiedrich et al., 2009 [e56] | Germany | Western Europe | 86/54 | ND/ND | ND/ND | 3/9 | No | Any exposure | Any class | Positive |  |
| Tanner et al., 2009 [e57] | USA | Northern America | 519/511 | 56/58 | 66/66 | 5/9 | No | Occupational | Any class | Positive |  |
| Richardson et al., 2009 [e58] | USA | Northern America | 50/43 | 70/49 | 65/71 | 5/9 | No | Any exposure | Insecticides | Positive |  |
| Firestone et al., 2010 [e59] | USA | Northern America | 404/526 | 62/62 | 69/71 | 7/9 | No | Occupational | Any class | Negative |  |
| Kiyohara et al., 2010 [e60] | Japan | Eastern Asia | 238/376 | 38/39 | 68/66 | 2/9 | No | Any exposure | Any class | Negative |  |
| Skeie et al., 2010 [e61] | Norway | Northern Europe | 212/175 | 59/59 | 70/67 | 5/9 | No | Any exposure | Any class | Positive |  |
| Weisskopf et al., 2010 [e62] | Finland | Northern Europe | 101/349 | 47/55 | 49/52 | 8/9 | No | Any exposure | Insecticides | Positive |  |
| Dutheil et al., 2010 [e63] | France | Western Europe | 207/482 | 57/59 | 69/69 | 5/9 | Yes | Any exposure | Insecticides | Positive |  |
| Vlajinac et al., 2010 [e64] | Serbia | Southern Europe | 110/220 | 57/57 | 60/60 | 4/9 | No | Any exposure | Herbicide, insecticide, fungicide | Positive |  |
| Sanyal et al., 2010 [e65] | India | Southern Asia | 175/350 | 80/ND | 55/55 | 4/9 | No | Occupational | Herbicide, insecticide | Positive |  |
| Pereira et al., 2010 [e66] | Portugal | Southern Europe | 88/176 | 66/ND | 64/ND | 4/9 | No | Any exposure | Any class | Positive |  |
| Manthripragada et al., 2010 [e67] | USA | Northern America | 351/363 | 57/49 | ND/ND | 4/9 | Yes | Any exposure | Insecticides | Positive |  |
| Gatto et al., 2010 [e68] | USA | Northern America | 333/336 | ND/ND | ND/ND | 2/9 | Yes | Any exposure | Herbicide | ND |  |
| Wang et al., 2011 [e69] | USA | Northern America | 362/341 | 57/52 | 68/67 | 3/9 | Yes | Any exposure | Herbicide and fungicide | Positive |  |
| Rugbjerg et al., 2011 [e70] | Canada | Northern America | 403/405 | 66/50 | 65/62 | 3/9 | No | Any exposure | Herbicide, insecticide, fungicide | Positive |  |
| Richardson et al. 2011 [e71] | USA | Northern America | 149/134 | 60/49 | 65/68 | 5/9 | No | Any exposure | Insecticide | Positive |  |
| Das et al., 2011 [e72] | India | Southern Asia | 345/370 | 62/59 | 62/62 | 5/9 | Yes | Occupational | Any class | ND |  |
| Lin et al., 2011 [e73] | Taiwan | Eastern Asia | 453/291 | 57/44 | 57/59 | 3/9 | No | Any exposure | Any class | ND |  |
| Tanaka et al., 2011 [e74} | Japan | Eastern Asia | 249/369 | 37/38 | 68/66 | 3/9 | No | Occupational | Herbicide and fungicide | Negative |  |
| Tanner et al., 2011 [e75] | USA | Northern America | 110/358 | 73/74 | ND/ND | 7/9 | Yes | Occupational | Herbicide, insecticide | Positive |  |
| Infante et al., 2011 [e76] | Spain | Western Europe | 237/203 | 54/40 | 65/73 | 3/9 | No | Any exposure | Any class | Positive |  |
| Goldman et al., 2012 [e77] | USA | Northern America | 87/343 | 72/76 | 61/61 | 7/9 | Yes | Occupational | Herbicide | Positive |  |
| Lee et al., 2012 [e78] | USA | Northern America | 357/754 | 57/47 | 68/66 | 3/9 | Yes | Any exposure | Herbicide | Positive |  |
| Kiyohara et al., 2013 [e79] | Japan | Eastern Asia | 238/368 | 57/47 | 68/66 | 3/9 | No | Occupational | Any class | Negative |  |
| Lee et al., 2013 [e80] | USA | Northern America | 287/440 | 38/38 | 69/67 | 3/9 | Yes | Any exposure | Insecticide | Positive |  |
| Pinhel et al., 2013 [e81] | Brazil | South America | 254/169 | 56/49 | 69/71 | 3/9 | No | Any exposure | Any class | Positive |  |
| Narayan et al., 2013 [e82] | USA | Northern America | 357/807 | ND/ND | 68/66 | 4/9 | Yes | Household | Insecticide | Positive |  |
| Longo et al., 2013 [e83] | Brazil | South America | 154/158 | 63/41 | 68/69 | 2/9 | No | Double | Any class | ND |  |
| Chhillar et al., 2013 [e84] | India | Southern Asia | 70/75 | ND/ND | 67/77 | 4/9 | No | Any exposure | Insecticide | Positive |  |
| Kamel et al., 2014 [e85] | USA | Northern America | 89/336 | 57/46 | 68/69 | 4/9 | Yes | Occupational | Insecticide, herbicide | Positive |  |
| van der Mark et al., 2014 [e86] | Netherlands | Western Europe | 444/876 | 63/64 | 68/68 | 5/9 | No | Occupationa | Herbicide, insecticide, fungicide | Positive |  |
| Fitzmaurice et al., 2014 [e87] | USA | Northern America | 354/518 | 57/48 | 68/67 | 2/9 | No | Any exposure | Herbicide, insecticide, fungicide | Positive |  |
| Wang et al., 2014 [e88] | USA | Northern America | 357/752 | 57/53 | 68/66 | 2/9 | No | Any exposure | Insecticide | Positive |  |
| Liew et al., 2014 [e89] | USA | Northern America | 357/750 | 75/73 | 68/66 | 4/9 | Yes | Occupational | Any class | Positive |  |
| Furlong et al., 2015 [e90] | USA | Northern America | 69/306 | 57/47 | ND/ND | 7/9 | Yes | Occupational | Insecticide, herbicide | Positive |  |
| Narayan et al., 2015 [e91] | USA | Northern America | 286/580 | 97/94 | 69/67 | 4/9 | Yes | Any exposure | Insecticide | Positive |  |
| Moisan et al., 2015 [e92] | France | Western Europe | 133/298 | 56/50 | 75/75 | 8/9 | Yes | Any exposure | Herbicide, insecticide, fungicide | Positive |  |
| Yang et al., 2015 [e93] | China | Eastern Asia | 88/88 | ND/ND | ND/ND | 7/9 | No | Any exposure | Any class | Positive |  |
| Beghdadli et al., 2016 [e94] | Algeria | Northern Africa | 321/322 | 100/100 | 66/66 | 6/9 | No | Any exposure | Any class | Positive |  |
| Paul et al., 2016 [e95] | USA | Northern America | 357/495 | 54/54 | 72/68 | 3/9 | Yes | Any exposure | Insecticides | Positive |  |
| Schirinzi et al., 2016 [e96] | Italy | Southern Europe | 300/300 | 57/49 | 70/69 | 6/9 | No | Any exposure | Any class | Positive |  |
| Biernacka et al., 2016 [e97] | USA | Northern America | 364/364 | 69/70 | 65/65 | 6/9 | No | Any exposure | Any class | Positive |  |
| Chuang et al., 2017 [e98] | Taiwan | Eastern Asia | 294/45,300 | 51/50 | ND/ND | 2/9 | No | Any exposure | Insecticide | Positive |  |
| Sanders et al., 2017 [e99] | USA | Northern America | 619/854 | 60/48 | 68/66 | 3/9 | Yes | Any exposure | Insecticide, herbicide | Positive |  |
| Brouwer et al., 2017 [e100] | Netherlands | Western Europe | 352/607 | 63/48 | 69/69 | 5/9 | Yes | Any exposure | Insecticide, herbicide, fungicide | Negative |  |
| Narayan et al., 2017 [e101] | USA | Northern America | 360/827 | 63/63 | 68/66 | 3/9 | Yes | Any exposure | Insecticide, herbicide, fungicide | Positive |  |
| Pouchieu et al., 2018 [e102] | France | Western Europe | 1,732/148,078 | 57/46 | ND/ND | 3/9 | Yes | Occupational | Herbicide, insecticide, fungicide | Negative |  |
| Rösler et al., 2018 [e103] | Egypt | Northern Africa | 416/445 | 58/55 | 58/48 | 3/9 | No | Any exposure | Any class | Positive |  |
| Paul et al., 2018 [e104] | USA | Northern America | 472/532 | 61/59 | 69/68 | 4/9 | No | Occupational | Herbicide and fungicide | ND |  |
| Tufail et al., 2019 [e105] | Pakistan | Southern Asia | 600/1200 | 61/50 | ND/ND | 3/9 | Yes | Any exposure | Any class | Positive |  |
| Goldman et al., 2019 [e106] | USA | Northern America | 103/357 | 81/75 | ND/ND | 7/9 | No | Occupational | Insecticide | Positive |  |
| Angelopoulou et al., 2019 [e107] | Greece | Southern Europe | 686/356 | 75/74 | 68/66 | 4/9 | Yes | Any exposure | Any class | Negative |  |
| Ventura-Chávez et al., 2019 [e108] | Mexico | Northern America | 60/144 | 59/37 | 62/ND | 3/9 | Yes | Any exposure | Any class | Negative |  |
| Georgiou et al., 2019 [e109] | Cyprus | Southern Europe | 229/462 | 48/63 | 64/62 | 4/9 | Yes | Any exposure | Any class | Negative |  |
| Calvo-Trujillo et al., 2019 [e110] | Colombia | South America | 16/43 | 55/50 | 71/71 | 3/9 | Yes | Any exposure | Insecticide and herbicide | Negative |  |
| Dardiotis et al., 2020 [e111] | Greece | Southern Europe | 104/110 | 50/53 | 71/70 | 4/9 | No | Any exposure | Insecticide | Positive |  |
| Silvestre et al., 2020 [e112] | Brazil | South America | 85/265 | 55/44 | ND/ND | 2/9 | Yes | Any exposure | Any class | Positive |  |
| Shrestha et al., 2020 [e113] | USA | Northern America | 491/65,619 | 64/38 | ND/ND | 3/9 | No | Occupational | Herbicide, insecticide, fungicide | Positive |  |
| Belvisi et al., 2020 [e114] | Italy | Southern Europe | 694/640 | 76/56 | 68/65 | 4/9 | Yes | Occupational | Any class | Positive |  |
| Poorrvliet et al., 2021 [e115] | Australia | Oceania | 1,334/1,127 | 59/55 | 74/74 | 1/9 | Yes | Any exposure | Any class | ND |  |
| Xu et al., 2022 [e116] | China | East Asia | 90/90 | 63/48 | 65/64 | 5/9 | Yes | Any exposure | Insecticide | Positive |  |
| Shermon et al., 2022 [e117] | USA | Northern America | 149/105 | 59/41 | 73/66 | 2/9 | Yes | Any exposure | Any class | Positive |  |
| Belingheri et al., 2022 [e118] | Italy | Southern Europe | 347/389 | 66/32 | 72/69 | 4/9 | Yes | Occupational | Any class | Positive |  |
| Payami et al., 2023 [e119] | USA | Northern America | 808/415 | 61/59 | 68/67 | 5/9 | Yes | Any exposure | Any class | Positive |  |
| Moura et al., 2023 [e120] | Brazil | South America | 562/374 | 63/30 | 61/60 | 3/9 | Yes | Household | Any class | Positive |  |
| Paul et al., 2024 [e121] | USA | Northern America | 1,653/824 | 52/44 | 67/65 | 3/9 | No | Any exposure | Herbicide | Positive |  |
| Kumar et al., 2024 [e122] | India | Southern Asia | 74/74 | 32/46 | 60/60 | 5/9 | Yes | Occupational | Insecticides | ND |  |
| Reynoso et al., 2024 [e123] | Global | Global | 18,819/545,751 | 62/62 | 73/73 | 3/9 | Yes | Household | Any class | Positive |  |
| Schaffner et al., 2024 [e124] | France | Western Europe | 71/147 | 60/44 | 68/68 | 6/9 | No | Any exposure | Any class | ND |  |

Abbreviations: PD, Parkinson’s disease; AAE, age at evaluation; ND, not described.

* Positive association between pesticide exposure and PD according to results reported by original articles.

The references cited in this Table can be found in Supplementary Table 6.
